# Molybdenum Nanofertilizer Boosts Biological Nitrogen Fixation and Yield of Soybean through Delaying Nodule Senescence and Nutrition Enhancement

**DOI:** 10.1021/acsnano.3c02783

**Published:** 2023-07-27

**Authors:** Mingshu Li, Peng Zhang, Zhiling Guo, Weidong Cao, Li Gao, Yuanbo Li, Chang Fu Tian, Qing Chen, Yunze Shen, Fazheng Ren, Yukui Rui, Jason C. White, Iseult Lynch

**Affiliations:** † College of Resources and Environmental Sciences, China Agricultural University, Beijing 100193, China; ‡ Department of Environmental Science and Engineering, University of Science and Technology of China, Hefei 230026, China; § School of Geography, Earth and Environmental Sciences, University of Birmingham, Edgbaston, Birmingham B15 2TT, United Kingdom; ∥ Institute of Agricultural Resources and Regional Planning, Chinese Academy of Agricultural Sciences, Beijing 100081, China; ⊥ State Key Laboratory for Biology of Plant Disease and Insect Pests, Institute of Plant Protection, Chinese Academy of Agricultural Sciences, Beijing 100193, China; # State Key Laboratory of Agrobiotechnology, College of Biological Sciences, China Agricultural University, Beijing 100193, China; ∇ National Key Laboratory of Human Factors Engineering, China Astronaut Research and Training Center, Beijing, 100094, China; ◆ Key Laboratory of Precision Nutrition and Food Quality, China Agricultural University, Beijing 100083, China; ¶ The Connecticut Agricultural Experiment Station, New Haven, Connecticut 06504, United States

**Keywords:** molybdenum disulfide nanoparticles, soybean, biological nitrogen fixation, nutritional quality, biotransformation

## Abstract

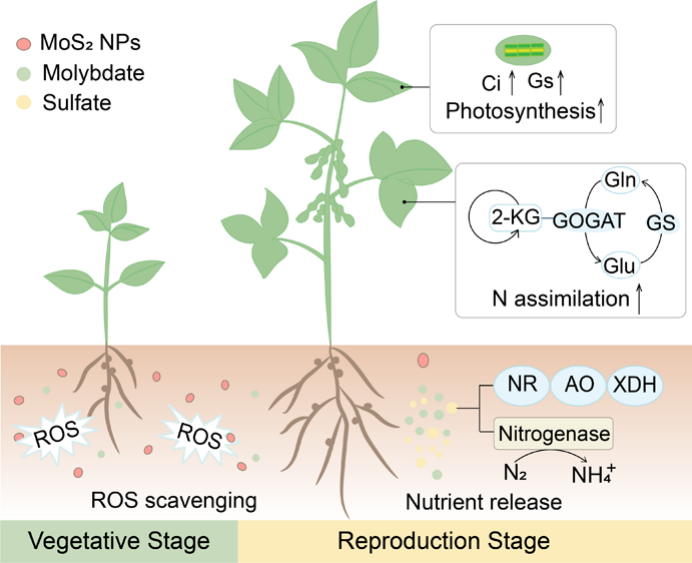

Soybean (*Glycine max*) is a crop of global significance and has low reliance on N fertilizers due to its biological nitrogen fixation (BNF) capacity, which harvests ambient N_2_ as a critical ecosystem service. BNF can be severely compromised by abiotic stresses. Enhancing BNF is increasingly important not only to alleviate global food insecurity but also to reduce the environmental impact of agriculture by decreasing chemical fertilizer inputs. However, this has proven challenging using current genetic modification or bacterial nodulation methods. Here, we demonstrate that a single application of a low dose (10 mg/kg) of molybdenum disulfide nanoparticles (MoS_2_ NPs) can enhance soybean BNF and grain yield by 30%, compared with conventional molybdate fertilizer. Unlike molybdate, MoS_2_ NPs can more sustainably release Mo, which then is effectively incorporated as a cofactor for the synthesis of nitrogenase and molybdenum-based enzymes that subsequently enhance BNF. Sulfur is also released sustainably and incorporated into biomolecule synthesis, particularly in thiol-containing antioxidants. The superior antioxidant enzyme activity of MoS_2_ NPs, together with the thiol compounds, protect the nodules from reactive oxygen species (ROS) damage, delay nodule aging, and maintain the BNF function for a longer term. The multifunctional nature of MoS_2_ NPs makes them a highly effective strategy to enhance plant tolerance to abiotic stresses. Given that the physicochemical properties of nanomaterials can be readily modulated, material performance (e.g., ROS capturing capacity) can be further enhanced by several synthesis strategies. This study thus demonstrates that nanotechnology can be an efficient and sustainable approach to enhancing BNF and crop yield under abiotic stress and combating global food insecurity.

## Introduction

Soybean is a vital crop that is rich in nutrients and serves as a significant source of vegetable protein for humans.^1^ The main source of N for soybean growth in intensive agriculture is biological nitrogen fixation (BNF), and nitrogen fertilizer, of which 40–80% is derived from BNF, will directly affect the yield and nutritional quality of soybean.^2^ BNF is also considered an effective approach to minimize agricultural carbon emissions by reducing energy-intensive nitrogen fertilizer inputs.^3^ However, BNF efficiency is widely limited by nutritional deficiencies, redox imbalance (excessive ROS) and high oxygen concentration.^4^ Additionally, extreme climates like drought, heat, and cold further complicate this situation as evidenced by the fact that the BNF rate is as low as 40% or less under abiotic stress.^5^

The past decades have witnessed substantial progress in BNF enhancement, largely driven by advancements in gene editing technologies and coincubation techniques involving beneficial microorganisms. The genomic revolution has led to the identification of numerous BNF genes, thereby enabling strategies to boost BNF through targeted gene editing.^6^ In this regard, gene editing has demonstrated outstanding potential in enhancing soybean BNF, achieving up to one times improvement in BNF efficiency.^7^ Alternatively, coinoculation of beneficial microorganisms with rhizobia has been shown to improve BNF rate and soybean yield and their tolerance to stress.^8,9^ Unfortunately, the broad effectiveness of these methods in agriculture remains elusive due to issues such as environmental heterogeneity and species variation. For example, the single trait change via GE may not be sufficient to support the effectiveness of BNF, especially when plants are exposed to abiotic stresses. Similarly, the overall efficacy of coincubation with beneficial microorganisms is highly dependent on plant variety and microbial species, and negative impacts are common.^10^ Therefore, practical application has been limited due to a lack of understanding of variation across soybean varieties or the timing of inoculation and planting. In addition, reduced effectiveness has been reported due to unsuitable environments, poor adaptation to the soil, and insufficient microorganism quality, among other issues.^10,11^

The cornerstone of efficient BNF lies in the enhancement of soybean nutrition and protection. Agriculture is currently experiencing a paradigm shift from intensification to precision and decarbonization. Nanotechnology has great potential as an innovative tool to enhance crop nutrition and protection, although an underlying mechanistic understanding of the observed results has often been elusive.^12,13^ For example, foliar application of Cu_3_(PO_4_)_2_ and CuO nanosheets suppressed *Fusarium virguliforme* induced soybean sudden death syndrome, with the hypothesized mechanisms being the release of antimicrobial Cu^2+^ and the stimulation of plant defense pathways by effective delivery of Cu.^14^ MoS_2_ enhanced the growth of rice through mechanisms such as promoting nitrogen source assimilation, enhancing metabolic reactions, and accelerating cell division and expansion.^15^

Similarly, foliar application of Fe_2_O_3_ NPs increased soybean BNF efficiency by regulating the antioxidant system and phytohormone.^16^ Separately, CeO_2_ nanoparticles protect plants from heat,^17^ high salinity,^18^ and nitrogen excess or deficiency;^19^ the proposed mechanism centers on ROS scavenging activity. Zhang et al. reported a similar mechanism for ROS responsive star polymers that were designed to enhance the tolerance of tomato seedlings to heat and light stresses.^20^ Therefore, we proposed that nanobiotechnology can provide an effective method to improve soybean BNF efficiency and yield by managing the redox balance and nutrients in the BNF environment, even under abiotic stress conditions.

As a two-dimensional material, MoS_2_ nanomaterials possess special physicochemical properties and a wide range of applications in fields such as medicine, electronics and energy.^21^ The excellent antioxidant enzyme mimicking activity of MoS_2_ nanomaterials has led to their application in cancer treatment.^22^ As an essential element for plants, Mo serves as the active center of nitrogenase and possesses immense potential for plant application, owing to its minimal dosage requirement and considerable economic returns. In the current study, we explored the potential of several MoS_2_ nanomaterial soil amendments to enhance BNF and soybean growth and tolerance to abiotic stress (drought and heat stress). The study was based on two hypotheses: (1) MoS_2_ NPs are bioavailable which release Mo and S in the soil–soybean–rhizobia system for the synthesis of Mo enzymes and S metabolites in soybean and rhizobia. (2) The well-known antioxidant enzyme mimicking activity of MoS_2_ will alleviate damage associated with excessive amounts of ROS^12^ that occurs in plant tissues under stress. These hypotheses were investigated in a soybean life cycle study, with the orthogonal evaluation of the key enzymes and genes involved in BNF and nitrogen assimilation, antioxidant systems, metabolomics, inorganic nutrient homeostasis, and Mo and S metabolism. We found time and materials type dependent release of Mo from the MoS_2_ to support nitrogen fixation and ROS capturing at different growth stages. This nanoscale-specific multifunctionality resulted in significantly enhanced BNF and yield (up to 35%, compared with the untreated control, and 30%, compared with molybdate treatment) with only a single low dose (10 mg/kg) of treatment at the early growth stage. Moreover, we found that the multifunctional properties of MoS_2_ NPs can be applied to other scenarios, such as enhancing the protection of soybean under extreme environmental conditions (such as drought and high temperature) by reducing oxidative stress and growth inhibition. This study demonstrates the high potential of MoS_2_ nanomaterials for promoting BNF and soybean yield to support efforts to combat global food insecurity under changing climates.

## Results and Discussion

### Characterization of Materials

**Figure 1:**
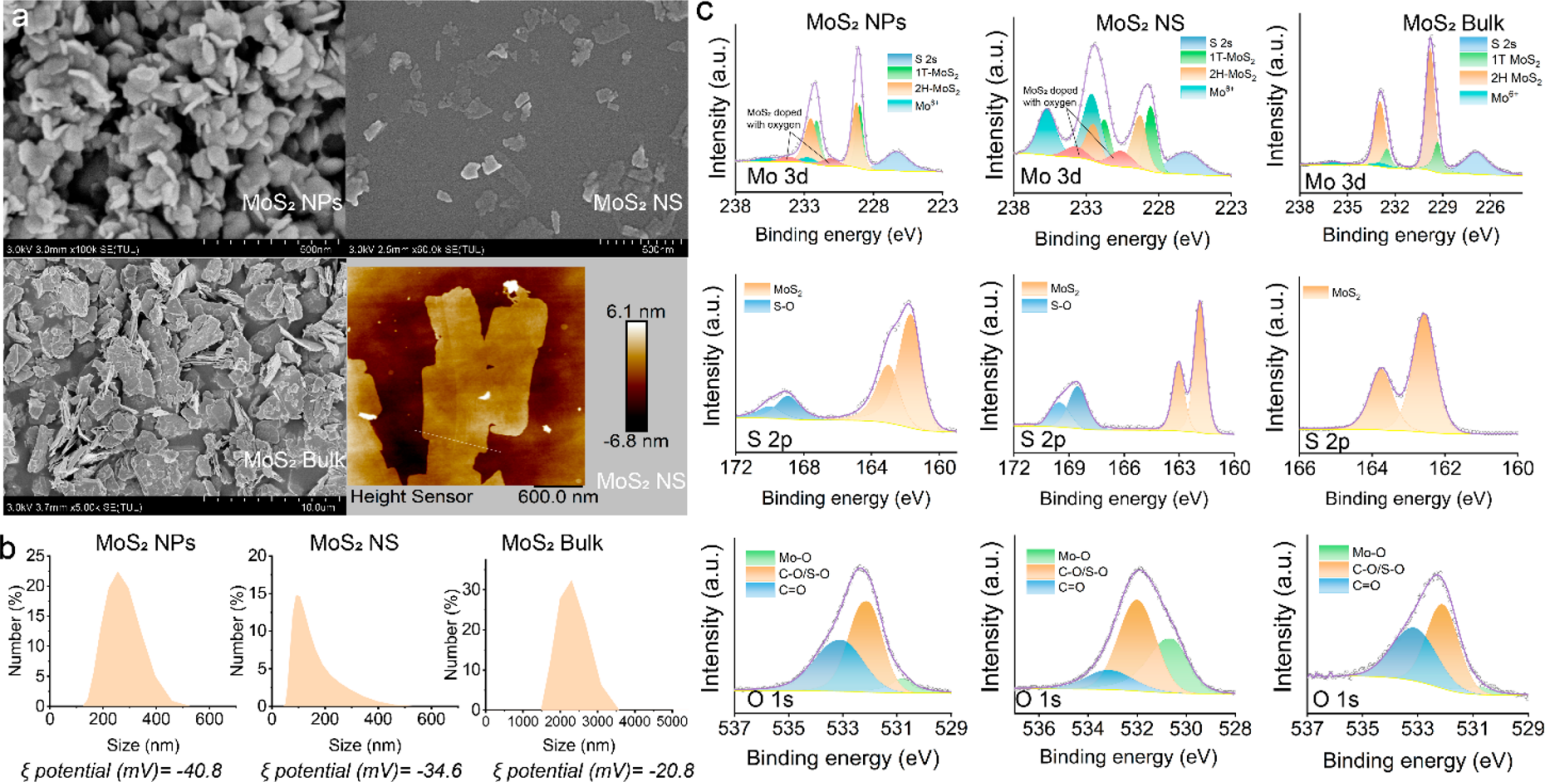
Morphology characterization of MoS_2_NPs, MoS_2_NS and MoS_2_Bulk. (a) SEM images of materials and AFM image of MoS_2_ NS. (b) Hydrodynamic size and surface charge analysis of materials. (c) X-ray photoelectron spectroscopy (XPS) of materials.

MoS_2_ exists in nature as the mineral molybdenite, which has a special layered structure similar to graphite. Differences in the lateral size and thickness lead to significant changes of physicochemical properties. Full characterization data of the materials are shown in Figure 1a. Scanning electron microscopy (SEM) revealed the morphology of MoS_2_ NPs, MoS_2_ NS, and MoS_2_ Bulk. The lateral size of the material was calculated using ImageJ software. The results show that the average lateral sizes of MoS_2_ NPs, MoS_2_ NS, and MoS_2_ Bulk are 106.8 nm, 115.6 nm, and 2.6 μm, respectively. The average thickness of the MoS_2_ NPs is 20.1 nm. Atomic force microscopy (AFM) images reveal that the thickness of the MoS_2_ NS is 4.3 nm (Figure 1a). The three types of MoS_2_ materials (50 mg/L) exhibited favorable dispersibility in deionized water, with respective zeta potentials of −40.8, −34.6, and −20.8 mV for MoS_2_ NPs, MoS_2_ NS, and MoS_2_ bulk. The corresponding hydrodynamic diameters were measured as 275.1 ± 8.38, 141.4 ± 3.56, and 3.543 ± 0.23 μm (Figure 1b).

Chemical structures of the MoS_2_ were analyzed by fitting the XPS spectra. The Mo(IV) 3d energy level of the three MoS_2_ materials exhibited primary peaks at around ∼232.3 and ∼229 eV, which correspond to 3d_3/2_ and 3d_5/2_, respectively, confirming the MoS_2_ structure (Figure 1c).^23^ The Mo(VI) 3d energy level of the three MoS_2_ materials exhibited primary peaks situated at ∼235.7 and ∼232.6 eV, corresponding to 3d_3/2_ and 3d_5/2_, respectively. The 1T phase of MoS_2_ NPs was confirmed by spectral features at 229 and 232.8 eV, while the 2H phase was identified by spectral features at 229.2 and 232.5 eV. MoS_2_ Bulk has the highest 2H to 1T ratio (3.5), followed by MoS_2_ NPs with a ratio of 1.44 and MoS_2_ NS with a ratio of 1. 1T-phase MoS_2_ exhibits increased solubility and oxidation rates compared to the 2H-phase and is more active in the environment.^24^ The Mo 3d spectra of MoS_2_ NPs and MoS_2_ NS showed the incorporation of O in MoS_2_. This phenomenon can be attributed to the higher electronegativity of oxygen compared to sulfur; when oxygen is incorporated into MoS_2_, it causes a shift in the binding energy of Mo 3d toward the high-energy region.^25^ In addition, the S–O 3d_3/2_ and S–O 3d_1/2_ NPs and MoS_2_ NS exhibited primary peaks situated at ∼168.5 and ∼169.8 eV, respectively. The fractions of the S–O component in MoS_2_ NPs and MoS_2_ NS were 17.7% and 45.4%. The O 1s spectrum of three MoS_2_ could be fitted into three peaks at around ∼530.7, ∼532.1, and ∼533.1 eV, corresponding to Mo–O bond, S–O/C–O bond and C–OH bond, respectively. The fractions of the Mo–O component in MoS_2_ NPs, MoS_2_ NS, and MoS_2_ Bulk were 10.9%, 34.8%, and 6.1%, respectively. These results showed that the presence of lattice oxygen in MoS_2_ NPs and MoS_2_ NS as well as oxygen-containing functional groups on the materials surface.^25^ It has been reported that O atom plays an important role in metal chalcogenide catalysts.^26^ The distinct properties of the three materials may result in different environmental behavior and phytoeffects, which will be examined next.

### Nanoscale MoS_2_ Improves Yield and Nutritional Quality of Soybean

**Figure 2:**
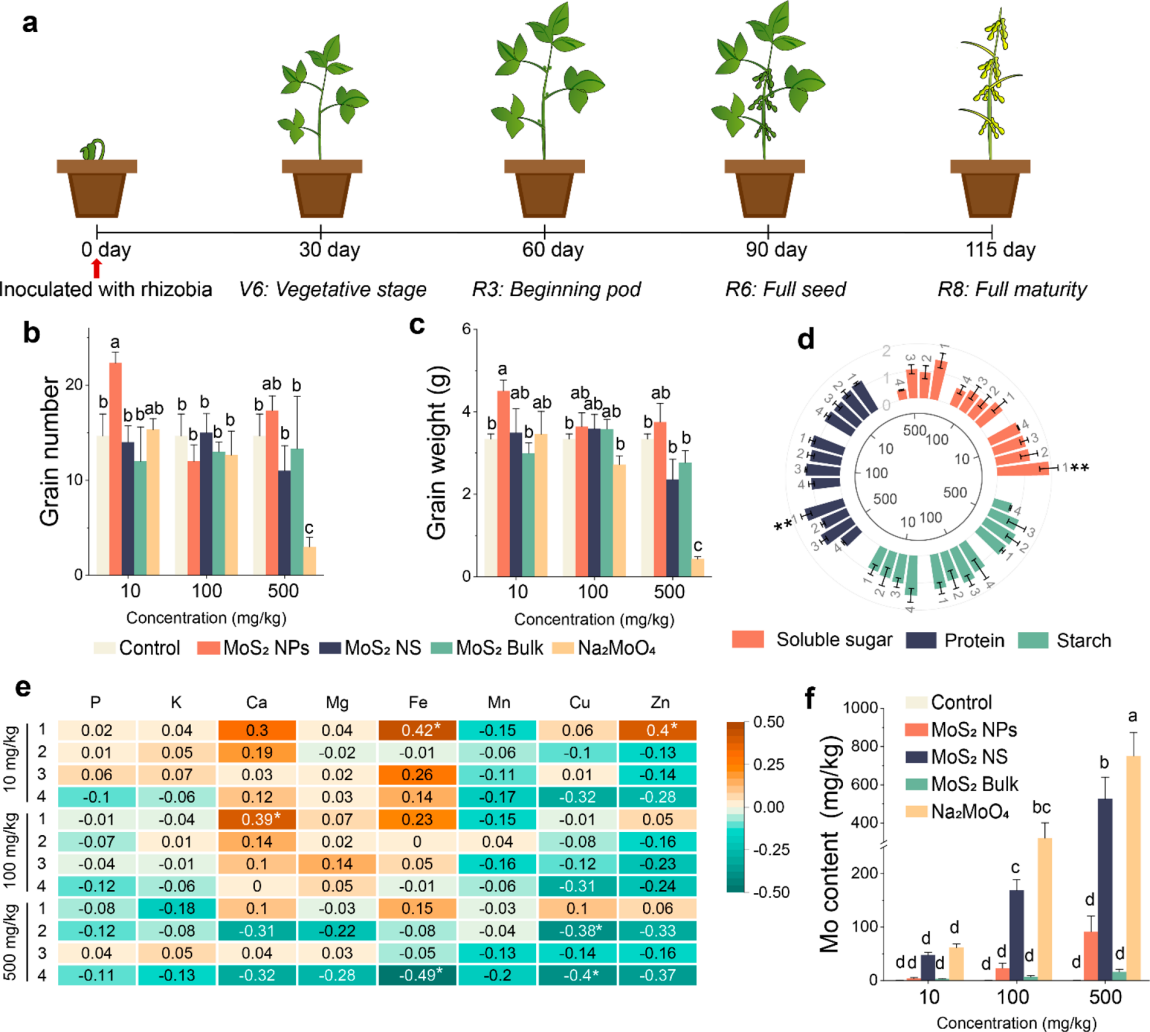
Yield and nutritional quality of soybean grains harvested at 115 days. (a) Schematic illustration of the growth stages of soybeans and sampling point. (b) Grain number. (c) Grain weight. (d) Content of organic nutrients. The bar is a fold change relative to that of the control group. The scale of the inner circle represents the concentration (mg/kg). (e) Contents of inorganic nutrients in soybean grain. In (d and e), the numbers (1, 2, 3, and 4) represent treatment groups MoS_2_ NPs, MoS_2_ NS, MoS_2_ Bulk, and Na_2_MoO_4_, respectively. (f) Mo content of grain. Statistical significance was tested with one way ANOVA analysis with Tukey’s test. The data are shown as the mean ± SD (*n* = 6). In (b, c, and f), different lowercase letters indicate significant difference between groups. In (d and e), * and ** represent significant differences compared with control at *P* < 0.05 and *P* < 0.01, respectively.

MoS_2_, along with sodium molybdate (Na_2_MoO_4_) fertilizer at doses of 10, 100, and 500 mg/kg, were applied in the soil. The soybean seedlings were transferred into the soil and inoculated with rhizobia. Plant growth and yield were then evaluated at various key stages of the life cycle (Figure 2a). MoS_2_ NPs at 10 mg/kg increased the grain number and weight by 46 and 30%, respectively, compared with conventional molybdate fertilizer, which has no effect at this dose (Figure 2b,c). The other Mo treatments at 10 or 100 mg/kg had no effect on grain yield. MoS_2_ NS and Na_2_MoO_4_ at 500 mg/kg reduced the yield. Similarly, Na_2_MoO_4_ reduced the grain number and weight by 80 and 87% at 500 mg/kg, respectively. Notably, MoS_2_ NPs at 500 mg/kg showed no negative effects on these parameters.

MoS_2_ NPs also improved the organic (Figure 2d) and inorganic nutritional contents (Figure 2e) of the grain. Specifically, MoS_2_ NPs increased the protein content by 46% at 500 mg/kg and increased the soluble sugar content by 91% at 10 mg/kg (Figure 2d). The remaining treatments had no overt effects on nutritional content, the exception being 500 mg/kg Na_2_MoO_4_ which dramatically decreased protein, starch, and soluble sugar content. MoS_2_ NPs at 10 mg/kg also significantly increased the content of Ca, Fe, and Zn by 30, 42, and 40%, respectively (Figure 2e); these elements are involved in pod development and are important to human nutrition.^27^ Ca content was also enhanced by 39% even at 100 mg/kg. However, MoS_2_ NS and Na_2_MoO_4_ at 500 mg/kg either had no effect or reduced the Ca, Fe, or Zn content. Notably, Mo adsorption in the grain is significantly less for MoS_2_ NPs than with MoS_2_ NS and Na_2_MoO_4_ (Figure 2f); although Mo toxicity is usually very rare, this does suggest reduced risk for the NPs form of the element.^28^ The health risk analysis further showed that Mo accumulated in soybean grains treated with 10 and 100 mg/kg MoS_2_ NPs posed no health risk to humans (Table S1).

These results suggest that MoS_2_ NPs are a more effective and likely safer approach to increase soybean yield and nutritional quality as compared to conventional Na_2_MoO_4_ fertilizer. The efficacy (35% increase of yield, compared with untreated control) is comparable to or higher than that reported for other approaches, such as gene editing (5–30%) or inoculation of microorganisms (3–15%) (Table S2).

### Nano-MoS_2_ Increase N Fixation and Assimilation in Plant

Soybean protein synthesis and growth are largely dependent on the uptake of nitrogen, which typically occurs by the combined effects of BNF and nitrogen fertilizer application in current agriculture. In the study presented here, no additional nitrogen fertilizer was added. Therefore, the enhanced yield and nutritional quality are only attributed to enhanced BNF. To verify this, we investigated the plant growth and biological processes linked to nitrogen assimilation at several key stages (30, 60, and 90 days) of the soybean life cycle.

We first examined the early stage (V6 stage, 30 days), which is a key period for establishing a symbiotic relationship between the soybean and rhizobia (i.e., the nodulation period). Both plant phenotype pictures and data (biomass and length) and photosynthetic parameters (relative chlorophyll content, P_n_, g_s_, C_i_, and T_r_) were either enhanced or unaffected by the MoS_2_ NPs treatment (Supplementary Figures 1–S3, see detailed results and discussion in the Supporting Information). Notably, nodule number and weight were increased significantly (Supplementary Figure 2e,f), suggesting enhanced BNF. This was further supported by the 20–36% increase of nitrogen uptake into the shoot tissues (Supplementary Figure 5d). However, the other treatments had no overt effects, and high doses of MoS_2_ NS and Na_2_MoO_4_ negatively impacted the photosynthetic system and reduced plant and nodule biomass (Supplementary Figures 2 and 3).

**Figure 3:**
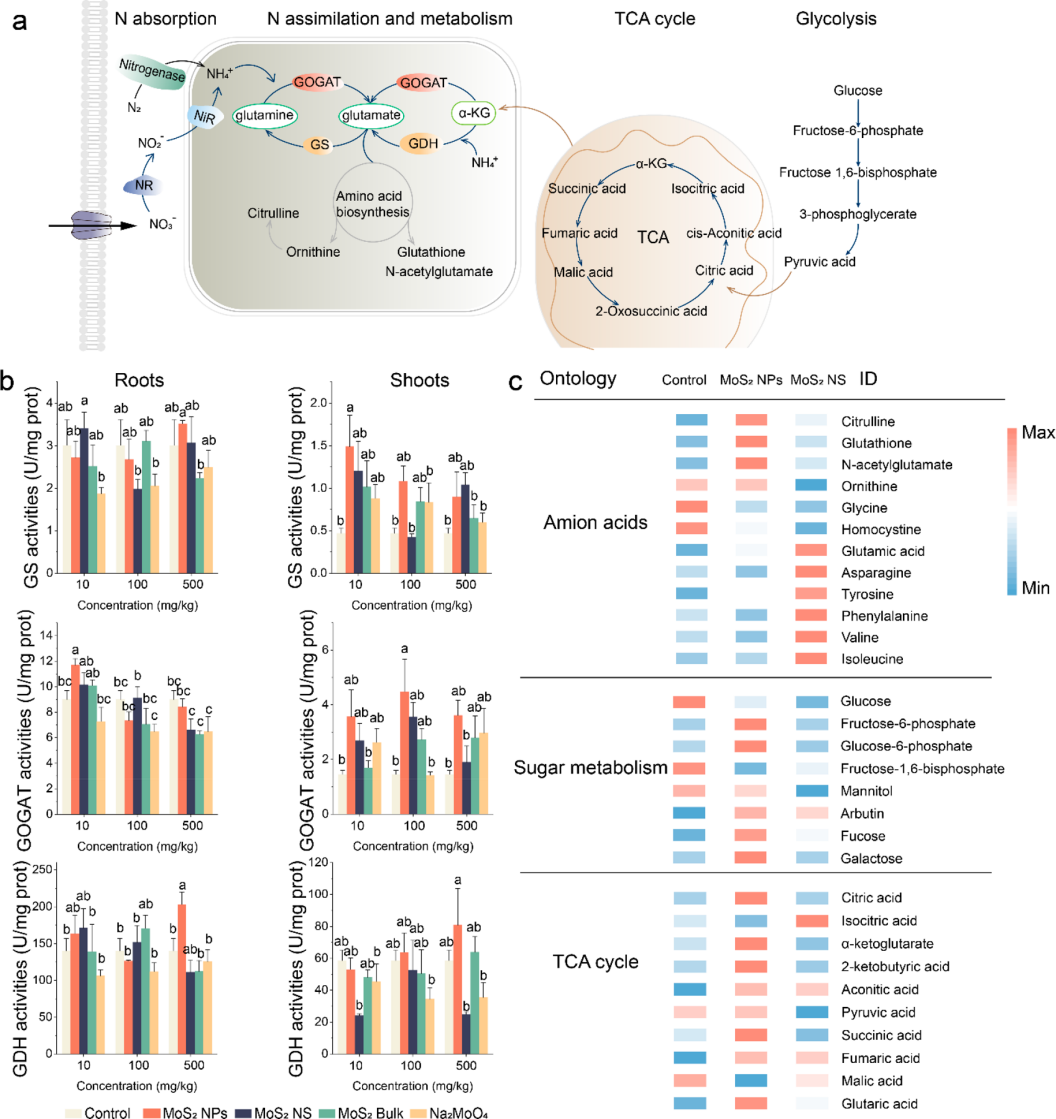
Molecular responses of nitrogen and carbon in soybean at 30 days. (a) Schematic diagram of carbon and nitrogen absorption, assimilation and metabolism. (b) Nitrogen assimilation-related enzymes, GS, GOGAT, and GDH activities in roots and shoots. (c) Heat map of amino acids, sugars, and TCA cycle metabolites in plants treated by 500 mg/kg MoS_2_ NPs and MoS_2_ NS, as well as the untreated control. In b, the data are shown as the mean ± SD (*n* = 6). Statistical significance was determined with one-way ANOVA analysis with Tukey’s test. Different lowercase letters indicate significant differences between groups.

Nitrogen uptake and assimilation involve several key enzymes, including glutamine synthetase (GS), glutamate synthase (GOGAT), and glutamate dehydrogenase (GDH). GS catalyzes ammonium and glutamic acid to produce glutamine, which is used for amino acid biosynthesis. GOGAT catalyzes glutamine and 2-oxoglutarate to produce glutamic acid. Glutamate dehydrogenase (GDH) catalyzes the reaction of NH_4_^+^ with 2-hydroxyglutarate to form glutamate, which is an alternative pathway for glutamate formation (Figure 3a).^29^ MoS_2_ NPs increased GOGAT activity by 0.3 times at 10 mg/kg and GDH activities in roots by 0.45-fold at 500 mg/kg (Figure 3b). MoS_2_ NPs increased the GS and GOGAT activity in the shoots by 2.17- and 1.45-fold at 10 mg/kg, respectively, and GOGAT activities by 2.08-fold at 100 mg/kg (Figure 3b), suggesting an enhancement of the GS-GOGAT cycle which could then accelerate nitrogen assimilation. Notably, increases were evident at all MoS_2_ NPs doses. However, the other Mo treatments showed much lower effects, and at the high dose, negative effects were again noted. The majority of products generated from nitrogen assimilation are utilized in photosynthesis, and enhancing nitrogen assimilation can stimulate the process of photosynthesis in plants.^30,31^ The nitrogen assimilation process of soybean was enhanced by MoS_2_ NPs, which promoted photosynthesis through a cascade reaction, thereby facilitating the growth of soybean during its nutritional and reproductive stages.

To confirm the results of the plant molecular regulation analysis, we tested the metabolomics of leaves treated with 500 mg/kg nanostructures (MoS_2_ NPs and MoS_2_ NS) by using GC-MS. Differential metabolites were analyzed by unsupervised clustering. Volcano plot analysis (unpaired *t* test with *P* < 0.05, fold-change > 1 and VIP > 1) identified differential metabolites in the MoS_2_ NPs and MoS_2_ NS treatment with control. Compared to MoS_2_ NPs, MoS_2_ NS at the high dose induced more downregulation (40 vs 22) and less upregulation of key metabolites (34 vs 44), indicating that the catabolic metabolism of plants is greater than the anabolic metabolism, resulting in a delay in the growth and development of the plants (Supplementary Figure 4d).^32^ The main enrichment pathway of MoS_2_ NPs and MoS_2_ NS treatment were related to amino acids and carbohydrates (Supplementary Figure 4b). Amino acids and carbohydrates directly reflect the accumulation and assimilation of nitrogen and carbon in plants, which indicates that MoS_2_ NS and MoS_2_ NPs affected soybean growth by regulating the assimilation of carbon and nitrogen. MoS_2_ NPs increased glutamate, glutathione, citrulline, and tyrosine by 1.32-, 1.23-, 2.31-, and 1.08-fold, respectively (Figure 3c). MoS_2_ NPs also increased the levels of α-ketoglutarate (α-KG), which is directly involved in nitrogen assimilation (Figure 3c), as well as the biomolecular precursors (i.e., glutaric acid and 2-ketobutyric acid). These molecular responses suggest that enhanced enzymatic activity and metabolite levels of the nitrogen assimilation system contributed to the enhanced nitrogen uptake by treated soybean (Supplementary Figure 5). The amino acids altered by MoS_2_ NS were mainly branched-chain and aromatic amino acids, which are essential for plants to cope with environmental stress, suggesting that MoS_2_ NS treatment re-established oxidative homeostasis in soybean. Sucrose is a source of energy and a precursor for biosynthesis in plants, while fructose-6-phosphate is a precursor for its synthesis. The decrease in ribose-5-phosphate and fructose-1,6-diphosphate and the increase in fructose-6-phosphate indicated that MoS_2_ NPs promoted the conversion of ribose-5-phosphate and fructose-1,6-diphosphate to fructose-6-phosphate. Therefore, MoS_2_ NPs accelerated sucrose synthesis and promoted soybean photosynthetic carbon assimilation. However, a high dose of MoS_2_ NS treatment downregulated the levels of galactitol, mannitol, rhamnose, and glucose in soybean leaves and upregulated trehalose-6-phosphate. Trehalose-6-phosphate can accelerate sucrose conversion to hexose and allow hexose phosphates to enter the central metabolic system, maintaining normal physiological metabolism of plants under carbon starvation.^33^ Therefore, it can be inferred that soybean treated with MoS_2_ NS was under carbon starvation. The TCA cycle supplements the carbon skeleton of the GS/GOGAT cycle and is a key process connecting carbon and nitrogen assimilation. MoS_2_ NPs increased the levels of TCA cycle-related metabolites, such as α-ketoglutarate, succinate, and citrate, indicating that MoS_2_ NPs upregulated the TCA cycle, which is closely related to plant biomass accumulation. In summary, MoS_2_ NPs promoted carbon and nitrogen assimilation in soybean evidenced by the up-regulation of amino acid and carbohydrate levels and the enhanced GS-GOGAT cycle.

### Biotransformation and Multifunctionality of Nano-MoS_2_

Mo is the metal center for nitrogenase, which catalyzes the BNF process, and for enzymes such as nitrate reductase (NR), aldehyde oxidase (AO) and xanthine dehydrogenase (XDH)^34^ that are involved in nitrogen assimilation, phytohormone synthesis, purine metabolism, and key oxidation–reduction reactions of nitrogen and sulfur metabolism.^35^

**Figure 4:**
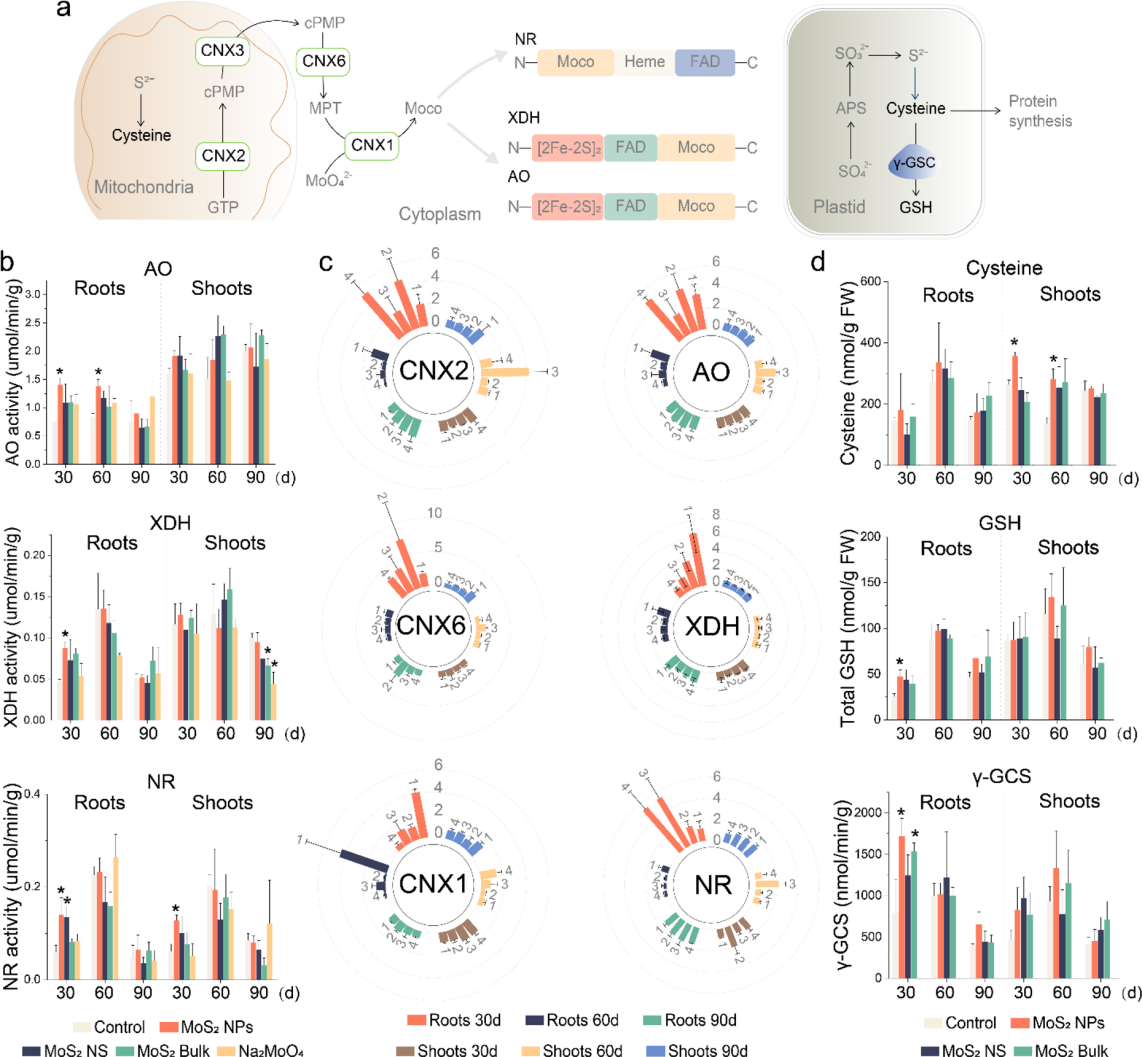
Effect of four materials on molybdenum enzymes in soybean. (a) Diagram of the synthesis of molybdenum cofactors and sulfur metabolites. (b–d) Effects of four materials at 500 mg/kg on molybdenum enzymes (b), the expression of molybdenum enzyme genes (c), and S metabolism (d) in soybean at 30, 60, and 90 days. In (d), the bar is a fold-change relative to the control group. 1, 2, 3, and 4 represent treatments of 500 mg/kg MoS_2_ NPs, MoS_2_ NS, MoS_2_ Bulk, and Na_2_MoO_4_, respectively. The data are shown as the mean ± SD (*n* = 6). Statistical significance was tested with one-way ANOVA analysis with a Tukey’s test. * represents *P* < 0.05.

The synthesis of Mo-cofactor starts in the mitochondria and is finalized in the cytoplasm (Figure 4a). Since soybean demand for nitrogen differs across various growth stages, we investigated the temporal dynamics of the activities of these enzymes and their regulating genes at 30 days, 60 days (R3 stage, beginning of podding, highest nitrogen demand), and 90 days (R6 stage, bulging period, nutritional growth stops, nitrogen demand is low) to further our mechanistic understanding (Figure 4b). MoS_2_ NPs increased AO and XDH activity in the roots at 30 days by 84 and 90%, respectively; AO activity at 60 days was still increased by 64%. NR activity in both roots and shoots was enhanced by 138 and 108%, respectively, after MoS_2_ NPs treatments. MoS_2_ NS also increased the NR by 129% at 30 days in roots. The effects of MoS_2_ bulk and Na_2_MoO_4_ were not significant, and at 90 days, treatment reduced XDH activities in shoots.

The modulation of these enzymes was further investigated at the genetic level (Figure 4c). The effects of Mo materials on gene expression were most evident in the roots in a time-dependent manner, with the strongest positive effects observed at 30 days. CNX1, CNX2, and CNX6 are proteins directly involved in the biosynthesis of molybdenum cofactors (Moco).^36^ At 30 days, all materials enhanced the expression of CNX2 and CNX6, both of which are involved in the initial steps of Moco synthesis (Figure 4c), with stronger effects observed for MoS_2_ NS and Na_2_MoO_4_. However, only MoS_2_ NPs increased the expression of CNX1 (310%), which mediates the final and essential step in Moco synthesis by inserting Mo into the proteins to achieve biological activity.^37^ Enhancement of CNX1 expression has been shown to increase NR and AO activities in soybean.^38^ This suggests that the increased expression of CNX1 played a key role because only MoS_2_ NPs increased its expression; this correlates well with the MoS_2_ NPs impact on enzyme activity. MoS_2_ NPs also significantly increased the expression of genes encoding the AO and XDH (238–541%). Other Mo materials also increased the expression of AO and NR; however, these increases did not directly correlate with the observed enzyme activities. Taken together, the results suggest that the Mo materials not only directly promote Moco by providing a Mo source but also regulate the gene expression of the key CNX proteins and Mo enzymes. The results also suggest the transformation of MoS_2_, because Mo can only be used and incorporated into Mo-enzymes in the MoO_4_^2–^ form; MoS_2_ NPs must dissolve and transform from Mo(IV) to Mo(VI) for plant use.

The transformation of MoS_2_ is further suggested by the alteration of the sulfur metabolism. Since sulfur is an essential macronutrient for plant growth, we hypothesize that sulfate released during oxidative dissolution can also be absorbed by plants and incorporated into the synthesis of sulfur containing biomolecules, including proteins and antioxidants (e.g., glutathione (GSH), Figure 4a). Therefore, we further quantified several important sulfur containing compounds including cysteine that is essential for protein synthesis, GSH which is an antioxidant, as well as γ-GCS which catalyze the GSH synthesis.^39^ MoS_2_ NPs significantly increased cysteine levels in shoots at 30 days (by 36%) and 60 days (107%), as well as GSH and γ-GCS (Figure 4d). However, effects of other treatments are not significant, except for MoS_2_ Bulk, which increased the γ-GCS by 39%.

**Figure 5:**
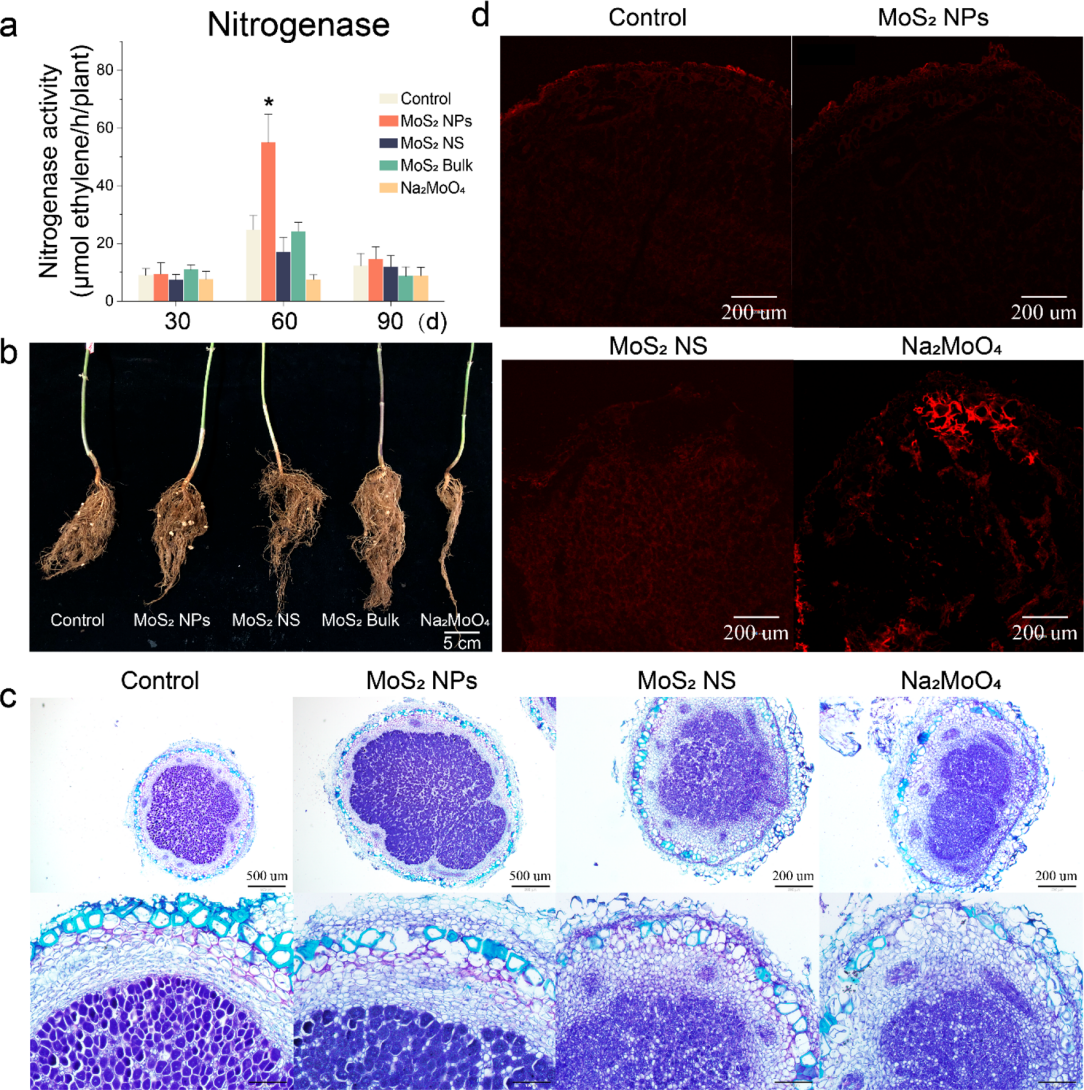
Effect of four materials on BNF in soybean. (a) Effects of four materials at 500 mg/kg on nitrogenase. (b) Phenotype of nodules treated by four materials at 500 mg/kg. **(**c) Paraffin-embedded sections of toluidine blue-stained nodules at 60 days. The picture below is a larger version of the picture on the right, and the scale is 100 μm. (d) Confocal microscope image of nodules (60 days) stained by ROS fluorescent probes.

We further examined the nitrogenase, which is directly responsible for N_2_ fixation. The activity of nitrogenase was largely unaffected by treatment, with the exception being that MoS_2_ NPs enhanced the activity by 122% at 60 days (Figure 5a). Moreover, MoS_2_ NPs significantly promoted the growth of the nodules (Figure 5b). Importantly, this is when the nitrogen demand of soybean is greatest, indicating enhanced BNF efficiency. At this stage, nodule aging has started, and the function of N_2_ fixation is declining. The nodules treated with MoS_2_ NPs had a significantly denser and deeper staining compared to the control, indicating that the infected cells had a higher symbiont density in the MoS_2_ NPs-treated nodules. Compared with the control, the symbiosis membrane was thinner in the infiltrated cells of the MoS_2_ NPs treatment group, which facilitated the BNF capacity (Figure 5c).^40^ On the other hand, the nodules treated with MoS_2_ NS and Na_2_MoO_4_ showed less rhizobial infestation and symbiont formation as demonstrated by the smaller and diffuse toluidine blue staining, indicating that BNF was significantly inhibited (Figure 5c). Nitrogenases are very sensitive to ROS, and reducing ROS in nodules is beneficial to BNF capacity.^41^ During nodule senescence, ROS accumulates excessively. We found that MoS_2_ NPs treatment significantly reduced the ROS accumulation in the nodule, while Na_2_MoO_4_ and MoS_2_ NS increased the ROS level (Figure 5d). Together with the optical morphology of the nodules, these results suggest that MoS_2_ NPs delayed the nodule senescence, thus maintaining the N_2_ fixation for a longer period.

**Figure 6:**
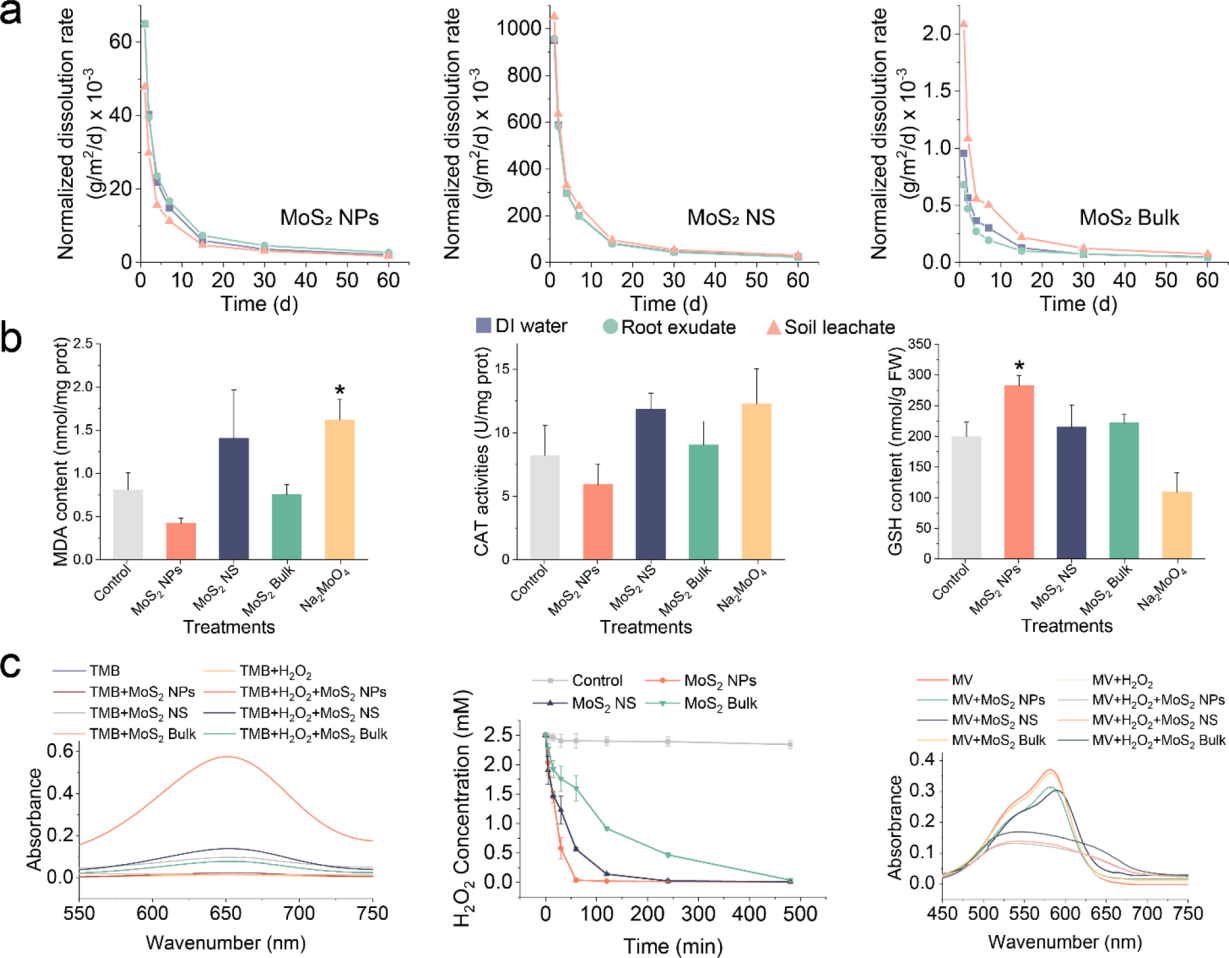
Mechanism based on the properties of the material. (a) Normalized dissolution rate (g/m^2^/d) of Mo from MoS_2_ NPs, MoS_2_ NS and MoS_2_ Bulk in DI water, root exudates, and soil leachate, based on the surface area of the materials. (b) Effects of four materials at 500 mg/kg on antioxidant system in nodules at 60 days. (c) Validation of the antioxidant-like enzyme activity of MoS_2_ NPs, MoS_2_ NS, and MoS_2_ Bulk. The data are shown as the mean ± SD (*n* = 6). Statistical significance was tested with one-way ANOVA analysis with a Tukey’s test. * represents *P* < 0.05.

The above-mentioned results suggest that MoS_2_ NPs not only improve the activity of Mo enzymes by regulating the gene expression of key CNX proteins and incorporating the Mo enzymes but also enhance the efficiency of BNF by reducing the accumulation of ROS in nodules. As discussed earlier, MoS_2_ must transform and release Mo so that Mo can be incorporated into the Mo enzymes. To further understand the transformation process, we calculated the measured BET surface area of the MoS_2_-normalized dissolution rates of MoS_2_ materials in different media including DI water, root exudates, and soil leachate over 60 days (Figure 6a). The amount of dissolved Mo was 1.3–3.8 mg/L for MoS_2_ NPs, 26.4–40.2 mg/L for MoS_2_ NS, and 0.016–0.09 mg/L for the MoS_2_ Bulk. The dissolution rates of the materials were normalized using their BET surface areas (Table S3). Results showed that the rate of MoS_2_ NPs (1.72 × 10^–3^–0.065 g/m^2^/days) was higher than that of MoS_2_ Bulk (4.36 × 10^–4^–3.08 × 10^–3^ g/m^2^/days) but significantly lower than that of MoS_2_ NS (0.023–1.05 g/m^2^/days). The results indicated that the MoS_2_ NPs are more stable than MoS_2_ NS and indicate that MoS_2_ NS exhibit the highest dissolution rate, while MoS_2_ Bulk is nearly insoluble and MoS_2_ NPs dissolve slowly, which enables the sustainable release of Mo and thus as well as the sulfur (Figure 6a). The findings presented herein align with the distinct properties of the materials, most notably in relation to MoS_2_ NS possessing the thinnest dimensions and the highest proportion of 1T phase, which attest to it high dissolution.^42,43^ In addition, the sustained release of Mo by MoS_2_ NPs can be considered as a crucial factor in the boost of Mo enzymes activity.

Since the oxidative stress was alleviated, which is another key mechanism for the enhanced N_2_-fixation and yield, we further investigated the effect of MoS_2_ on the antioxidant system of soybean nodules (60 days). The nodules treated with MoS_2_ NPs had the lowest MDA content, indicating a reduction in ROS accumulation in nodules compared to the control, MoS_2_ NS, and Na_2_MoO_4_ groups, which was in accordance with the ROS fluorescence distribution results (Figures 5d and 6b). ROS can damage nodules, reduce nitrogenase activity, and hasten senescence. This finding suggests that MoS_2_ NPs protected nodule from ROS damage and delay the nodule senescence. However, MoS_2_ NPs did not trigger significant change of the activities of antioxidant enzymes such as CAT and POD in the nodule, suggesting that MoS_2_ NPs protected the nodule mainly by capturing the ROS scavenging mechanism rather than regulating the enzymatic antioxidant system. This was further demonstrated by comparing the ROS capturing capacities of the three materials (Figure 6c). The antioxidant enzyme can oxidize TMB to oxTMB (blue) with the maximum absorption peak at 652 nm.^44^ In our study, TMB was oxidized by MoS_2_ NPs, MoS_2_ NS, and MoS_2_ Bulk, with the highest oxidation of TMB in the MoS_2_ NPs system (Figure 6c). The H_2_O_2_ measurements showed that MoS_2_ NPs decomposed almost all of the H_2_O_2_ within 1 h, while MoS_2_ NS and MoS_2_ Bulk achieved similar results at 240 and 480 min, respectively (Figure 6c). The amount of ·OH was assessed by detecting the degree of ·OH-induced discoloration of methyl viologen.^45^ In the absence of H_2_O_2_, the absorbance of MoS_2_ NPs and MoS_2_ NS systems was clearly lower than that of MV, while the absorbance of MoS_2_ Bulk was comparable to that of MV, suggesting that the degradation of MV catalyzed by the electron transfer of MoS_2_ NPs and MoS_2_ NS may have occurred. In the presence of H_2_O_2_, all three materials significantly reduced the absorbance of the system, with MoS_2_ NS and MoS_2_ NPs reducing it more than MoS_2_ Bulk. These results indicate that both MoS_2_ NPs and MoS_2_ NS have ROS capturing activity, which agrees with the XPS results (Figure 1c). Specifically, the high percentage of 1T in these materials and the oxygen doping enable them to exhibit excellent catalytic properties.^46,47^ However, the high dissolution rate of MoS_2_ NS in the soil–plant system reduces benefits as a ROS scavenger and directly causes cytotoxicity. MoS_2_ NPs can capture ROS, protecting sensitive tissues from damage and contributing to delayed aging and prolonged functionality of nodules. Collectively, these data support our hypotheses that MoS_2_ NPs are a multifunctional amendment that can improve BNF and soybean yields by several mechanisms.

### Nano-MoS_2_ Enhances Tolerance to Abiotic Stress

**Figure 7:**
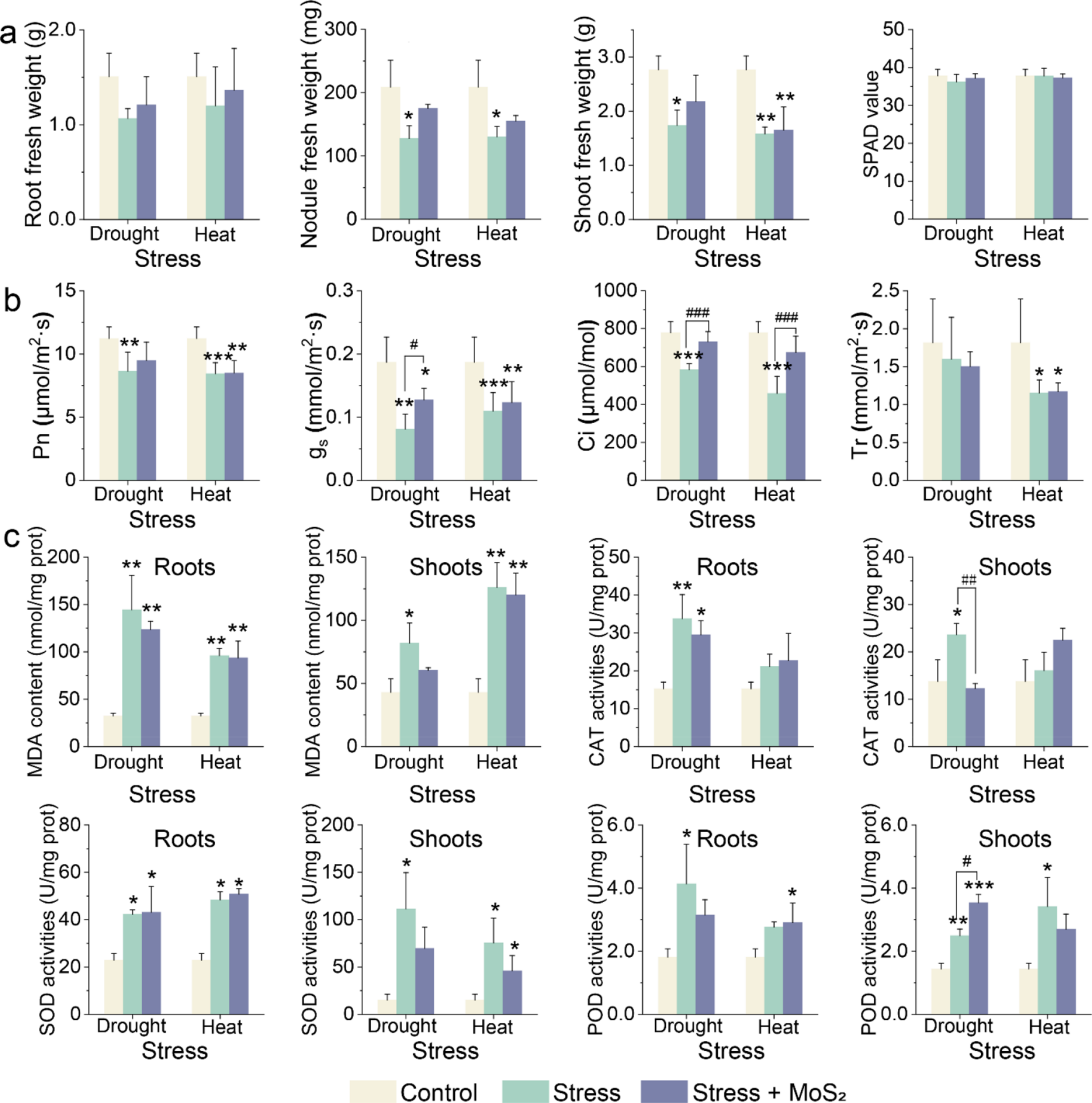
Ability of MoS_2_NPs to improve plant resistance to abiotic stresses. (a) Phenotype parameters: Fresh weight of roots, shoots, and nodules. (b) Photosynthetic parameters: P_n_, g_s_, C_i_, and T_r_. (c) Oxidative stress indicators: MDA content and CAT, SOD, and POD activities of soybean roots and shoots. The data are shown as the mean ± SD. Statistical significance was tested with one-way ANOVA analysis with a Tukey’s test. * represents *P* < 0.05, ** represents *P* < 0.01, and *** represents *P* < 0.001 compared with control. #, ##, and ### represent *P* < 0.05, *P* < 0.01, and *P* < 0.001 between the groups, respectively.

The use of the ROS capturing mechanism was further explored to help soybean plants cope with abiotic stress. Excessive ROS accumulation from stress related oxidative bursts is a major cause of plant death under stresses; MoS_2_ NPs demonstrated a clear potential to enhance soybean tolerance to abiotic stress by capturing the ROS and providing essential Mo and S nutrients. Soybean phenotype (biomass and plant height) and photosynthetic output were significantly improved by MoS_2_ NPs (Figure 7a,b; see detailed results and discussion in the Supporting Information). The level of oxidative damage was also reduced, as demonstrated by the reduced MDA levels and antioxidative responses (Figure 7c). The superior ROS scavenging ability of MoS_2_ NPs protects plants from damage (e.g., nodule aging) and also releases Mo and S that can be readily incorporated into several key physiological processes, including BNF and antioxidant processes.

## Conclusions

**Figure 8:**
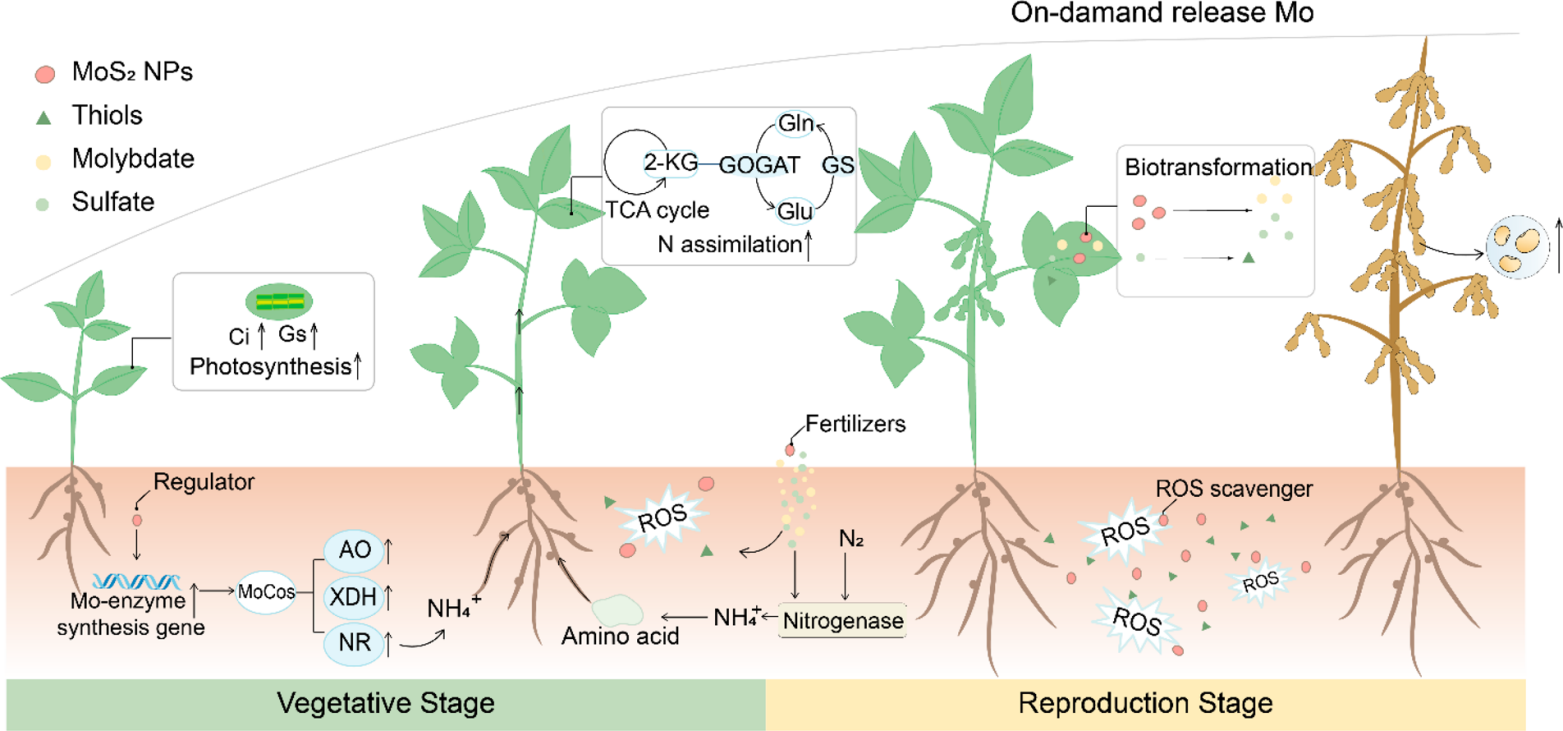
Schematic illustration of the multifunctionality of MoS_2_NPs. MoS_2_ NPs can release Mo in a responsive fashion to support BNF and capture the ROS at different growth stages to promote soybean C and N assimilation. In the early stage (vegetative) of soybean, young seedlings are usually vulnerable to stress. The majority of the MoS_2_ NPs remain as intact particles, so they can function as enzymes to capture the ROS while releasing a small portion of Mo to support nodule formation and N_2_-fixation. At a later stage (reproductive), soybean needs a large amount of nitrogen nutrients, while the nodule function also starts to decline due to nodule senescence. The MoS_2_ NPs continuously dissolve and release more Mo to support N_2_-fixation. While part of the nanoenzymes still remain as intact particles and maintain the enzyme mimetic function, they can protect the nodule cells and maintain their N_2_-fixation ability by capturing the ROS and delaying nodule senescence.

In summary, we report that MoS_2_ NPs can be used as a nanofertilizer to enhance BNF and soybean yields through multifunctional mechanisms (Figure 8). The addition of a single low dose to soil at the beginning of the season can increase the soybean yield by 35% while simultaneously increasing seed nutrition (i.e., biofortification). The increase of yield is comparable to or higher than those that have been reported using genetic modification (GMO) or rhizobia inoculation methods for improving BNF (Table S2). The application strategy is practical and easy for farmers. Unlike the GMO method, which is affected by plant species and type of stressors, the mechanisms here are based on the material properties and thus have the potential to be used to enhance plant tolerance under various abiotic stresses. The results of this study demonstrate the potential of nanotechnology for enhancing food security while reducing the input of chemical fertilizers into the environment. The difference among the three types of MoS_2_ suggests that the materials might be further optimized to enhance their efficacy.

## Methods

### Greenhouse Experiment

The soil was collected from an agricultural field in Beijing (40°14′40.91″ N; 116°19′17.94″ E) and mixed with potting soil (Scotts Miracle-Gro Products Inc., USA) at a volume ratio of 1:1. The properties of the mixed soil are listed in Table S4. MoS_2_ NPs, MoS_2_ NS, MoS_2_ Bulk, or Na_2_MoO_4_ were mixed with the soil thoroughly in plastic pots to achieve final concentrations of 10, 100, and 500 mg/kg; untreated soil was used as a control. Soybean seedlings (5 days old) of uniform size were transferred into the pots. A rhizobia (*Sinorhizobium fredii*) solution (1 mL, OD_600_ = 0.2) was injected into each pot to initiate nodulation. The seedlings were then placed in a greenhouse at Chinese Agricultural University with a day/night temperature of 25 °C/25 °C and a humidity of 70%. Details on seed germination and plant cultivation can be found in the Supporting Information Section 1. The plants were harvested at different growth stages (30, 60, 90, and 115 days) for different end points and analyses.

At 30 days post-treatment (V6 stage), seedlings were divided into shoots, roots, and nodules. The biomasses and lengths of roots and shoots, several photosynthetic parameters, inorganic nutrient contents, antioxidant activities, and metabolomic profiles were measured to evaluate the plant response at the early growth stage. At day 115 (R8 stage, full maturity), soybean seeds were harvested to determine yield as well as organic and inorganic nutritional quality. To determine the mechanism of the action of MoS_2_ and the difference between the different Mo materials, key enzymes involved in nitrogen fixation and assimilation and associated genes were quantified across the three key growing stages (V6, R3, and R6 stages). The dynamic adsorption and biotransformation of MoS_2_ materials were determined by measuring the Mo and S content and chemical species in both plant tissues and soil using orthogonal techniques including single particle inductively coupled plasma mass spectrometry (sp-ICP-MS) and synchrotron radiation-based X-ray fine structure spectroscopy (XAFS). Full details of the analytical methods are described below and in Supporting Information Section 1.

### Photosynthesis Measurement

Photosynthetic efficiency including the net photosynthesis rate (P_n_), stomatal conductance (g_s_), intercellular carbon dioxide concentration (C_i_), and transpiration rate (T_r_) was measured by an open gas exchange system (LI-COR Biosciences, Lincoln, NE) (Supporting Information Section 1). The relative chlorophyll content at 10 points near the main vein of the same leaf was measured using a SPAD-502 Plus (Konic Minolta, Japan).

### Organic Nutrient Analysis

The soluble protein content was determined using a total protein quantitative assay kit (Nanjing Jiancheng Co., Nanjing, China) according to the manufacturer’s instructions. The soluble sugar and starch contents were determined by the anthrone colorimetry method. The details of the analysis are described in Supporting Information Section 1.

### Elemental Analysis

Freeze-dried plant samples were ground into fine powders and digested in a mixture of nitric acid and hydrogen peroxide (v/v: 3:1) in a microwave digestion system (MARS 6, UK). Elemental content (Mo, S, Fe, Zn, Mn, Mg, Cu, P, Ca, and K) was then determined by ICP-MS (Thermo Scientific). Shoot tissues (GBW 07602) were used as standard reference materials as described by Zhang et al.^48^ Calibration standards of known concentrations (0.01–100 ppm) were used for quantification. The element recovery rates are presented in Table S5.

### Enzymes Involved in Nitrogen Fixation and Assimilation

The activities of GS, GOGAT and GDH were determined according to Wang et al.^29^ AO and XDH activities were measured according to the method described by Nie et al.^49^ NR activity was determined followed Su et al.^50^ Nitrogenase activity was measured using an acetylene reduction assay (ARA). Details of the analytical procedures were provided in Supporting Information Section 1.

### Enzymatic and Nonenzymatic Antioxidants

Antioxidant enzymes including CAT, POD and SOD, and nonenzyme antioxidants including cysteine, GSH/GSSG and γ-GCS in roots and shoots were measured using the commercial assay kits (Nanjing Jiancheng Co., Nanjing, China) based on the manufacturer instructions.

### Quantitative Real-Time PCR Analysis

To analyze the expression of genes related to the synthesis of Moco and molybdenum enzymes including CNX1, CNX2, CNX3, AO, XDH, and NR, fresh roots and shoots were ground to a fine powder in liquid nitrogen. Total RNA was extracted using TRIzol Reagent (Invitrogen, USA). The RNA concentration was determined by a Nano-Drop2000 spectrophotometer (Thermo, USA) and cDNA was synthesized using 20 μg of RNA and SuperScript III RNase H–Reverse Trancrip-tase (Invitrogen, USA) according to the manufacturer instructions. The SYBR Premix Ex TaqTM Kit (TaKaRa, Dalian, China) and the Light Cycler System (Bio-Rad, Richmond, CA) were used for RT-PCR. The actin gene was used as the internal standard. Primer sequences for each gene are shown in Table S6.

### Metabolite Extraction and Analysis

Metabolomic analysis was performed on soybean shoots treated with 500 mg/kg Mo fertilizers for 30 days. Fresh samples (100 mg) were ground into a powder in liquid nitrogen and added to 80% methanol. The mixtures were ultrasonicated at ambient temperature for 30 min. A chloroform/deionized water (1:2, v/v) mixture (600 μL) was added to the samples, followed by vortexing and sonication at 25 °C for 30 min and centrifugation at 12,000 rpm for 10 min at 4 °C for GC-MS analysis. The measurement parameters and data analysis methods are presented in Supporting Information Section 1.

### Dissolution Experiment

The release of Mo ions from MoS_2_ NPs, MoS_2_ NS, and MoS_2_ Bulk was investigated by incubating the materials in root exudates, soil leachate, and deionized water over a course of 60 days. Briefly, MoS_2_ NPs, MoS_2_ NS and MoS_2_ Bulk were added into the media at 100 mg/L. All solutions were sonicated for 1 min and placed in a thermostat at 25 °C in the dark. Three replicate samples were collected on days 1, 2, 4, 7, 15, 30, and 60. The particles are removed from the sample by a centrifugal ultrafiltration unit (3 kDa MWCO tubes, Millipore, Amicon Ultra). The samples were acidified by 3% HNO_3_ for measurement of Mo content using ICP-MS.

To understand the dissolution of MoS_2_ in soil with plants, the three forms of MoS_2_ and Na_2_MoO_4_ were mixed with the soil at 100 mg/kg. Soil pore water samples were collected every 15 days for analysis of Mo content using ICP-MS. Details of the extraction of root exudates, soil leachates, and pore water are provided in Supporting Information Section 1.

### Validation of Antioxidant-like Enzyme Activity

A 50 μL portion of 8 mM H_2_O_2_ solution was mixed with 50 μL of 1 mg/mL MoS_2_ NPs, MoS_2_ NS, and MoS_2_ Bulk solutions, respectively. The concentration of H_2_O_2_ in the solutions was measured at 5, 15, 30, 60, 120, 240, and 480 min. 1 mg/mL of MoS_2_ NPs, MoS_2_ NS, and MoS_2_ Bulk solutions were mixed with TMB, MV, TMB+H_2_O_2_, and MV+H_2_O_2_ solutions, respectively. After incubation for 30 min, the catalytic ability of NMs was assessed using UV–vis absorption spectra in spectral and band scan mode.

### Statistical Analysis

The greenhouse experiment was a completely randomized design with six replicates of each treatment. Values are shown as the mean ± SD. Statistical analysis was performed on SPSS 19.0. Statistical significance was evaluated through one-way ANOVA. The mean values of each treatment were compared using the Turkey test. *P* < 0.05 was represented significantly different.

## Supporting Information Available

Details of the experimental methods: nanomaterial characterization, seed germination and plant culture, biochemical analyses, metabolomics analysis, microscopic observation, collection of root exudates, soil leachate and soil pore water, and pot experiment on drought and heat stress; details of supplementary data, results, and discussion: human health risk evaluation of soybean grain, effects of Mo fertilizers on the soybean growth, photosynthesis, C/N accumulation and metabolic profiles (30 days data), effects of Nano-MoS_2_ on the soil microbes, and nano-MoS_2_ as an enhancer of plant tolerance to abiotic stress, health risk assessment of soybean grains, current approaches to improve the efficiency of soybean production, soil parameters used in the experiment, limit of detection, precision and recovery data of ICP-MS for the selected elements, and primer sequences used for RT-PCR analysis (PDF)
