# Supplementary material for: Molybdenum Nanofertilizer Boosts Biological Nitrogen Fixation and Yield of Soybean through Delaying Nodule Senescence and Nutrition Enhancement
Source: ACS Nano. 2023 Jul 27;17(15):14761–74. doi: 10.1021/acsnano.3c02783 (PMC10416561; doi:10.1021/acsnano.3c02783)
Supplement: Supplementary file 1 — nn3c02783_si_001.pdf [file nn3c02783_si_001.pdf]

Supplementary Information

Molybdenum nanofertilizer boosts biological nitrogen fixation and yield of soybean through delaying nodule senescence and nutrition enhancement

Mingshu Li[1,2,†], Peng Zhang[2,3,†,*], Zhiling Guo[3], Weidong Cao[4], Li Gao[5,*], Yuanbo Li[1], Chang Fu Tian[6], Qing Chen[1], Yunze Shen[7], Fazheng Ren[8], Yukui Rui[1,*], Jason C. White[9,*], Iseult Lynch[3]

[1]College of Resources and Environmental Sciences, China Agricultural University, Beijing 100193, China

[2]Department of Environmental Science and Engineering, University of Science and Technology of China, Hefei 230026, China

[3]School of Geography, Earth and Environmental Sciences, University of Birmingham, Edgbaston, Birmingham, B15 2TT, UK

[4]Institute of Agricultural Resources and Regional Planning, Chinese Academy of Agricultural Sciences, Beijing 100081, China

[5]State Key Laboratory for Biology of Plant Disease and Insect Pests, Institute of Plant Protection, Chinese Academy of Agricultural Sciences, Beijing 100193, China

[6]State Key Laboratory of Agrobiotechnology, College of Biological Sciences, China Agricultural Bijing 100193, China

[7]National Key Laboratory of Human Factors Engineering, China Astronaut Research and Training Center, Beijing, 100094, China

[8]Key Laboratory of Precision Nutrition and Food Quality, China Agricultural University, Beijing 100083, China

[9]The Connecticut Agricultural Experiment Station, New Haven, CT 06504, United States

† These authors contributed equally.

*Corresponding authors: p.zhang.1@bham.ac.uk (P.Z.); gaoli03@caas.cn (G.L.);
Jason.White@ct.gov (J.C.W); ruiyukui@163.com (Y.R.)

Contents

Section 1. Supplementary methods 1

Chemicals and nanomaterial characterization. 1

Seed germination and plant culture 1

Photosynthesis measurement 2

Soluble sugar and starch contents measurement 2

Enzymes involved in nitrogen fixation and assimilation. 3

Metabolomics analysis 5

Optical microscope and confocal laser scanning microscope observation 5

Collection of root exudates, soil leachate and soil pore water 6

Pot experiment on drought and heat stress 6

Section 2. Supplementary data, results and discussion. 7

Human health risk evaluation of soybean grain 7

Effects of Mo fertilizers on the soybean growth, photosynthesis, C/N accumulation and metabolic profiles (30 days data) 8

Effects of Nano-MoS_2 on the soil microbes 14

Nano-MoS_2 as an enhancer of plant tolerance to abiotic stress 16

Section 3. Supplementary Table 19

Table S1 19

Table S2 19

Table S3 20

Table S4 20

Table S5 20

Table S6 20

References: 21

Section 1. Supplementary methods

Chemicals and nanomaterial characterization.

The MoS_2 NPs (99.99%, 80-100 nm) was obtained from Guangdong Nanuo Materials Technology Co., Ltd (Guangdong, China). MoS_2 Bulk, Na_2MoO_4 and all the other chemicals are purchased from Millipore Sigma. MoS_2 NS synthesized using previously reported methods[1, 2]. The MoS_2 (Millipore Sigma, China) was added to a hexane solution of 1.6 M n-butyllithium and stirred for 48 h under a stream of nitrogen. The product was then washed 3 times with hexane to remove lithium reagents and by-products. The product was sonicated for 1 h and then the unexfoliated MoS_2 material was removed by centrifugation at 500 rpm for 10 min to obtain a well-dispersed MoS_2 nanosheet dispersion. LiOH was removed from the dispersion by dialysis. The morphology and primary size of MoS_2 NPs, MoS_2 NS and MoS_2 Bulk were characterized by scanning electron microscopy (SU8020, HITACHI, Japan). The zeta potential and hydrodynamic sizes of the MoS_2 materials in deionized water (50 mg/L) were analyzed with a Zetasizer (Nano ZS90, Malvern, UK). The thickness of MoS_2 NS was analyzed by atomic force microscopy (Dimension Icon, Bruker AXS, Germany). The phase composition of MoS_2 NPs, MoS_2 NS and MoS_2 Bulk were determined by X-ray photoelectron spectroscopy (ESCALAB 250Xi, Thermo Scientific, USA) equipped with a monochromatic Al–Kα X–ray source (1486.6 eV).

Seed germination and plant culture

Soybean seeds (Hedou 13) purchased from Shouguang Seeds & Seedling Co., Ltd were sterilized with 5% (v/v) H_2O_2 for 5 minutes and rinsed with deionized water. Seeds were placed on filter paper which was soaked with deionized water in the tray and germinated in an incubator at 25 °C in the dark for five days. Subsequently,

soybean seedlings with uniform sizes were selected and each seedling was carefully planted in the mixed soil. The physiochemical properties of the soil are shown in Supplementary Table 1. Each treatment sample was watered every two days with 150 mL. After 90 days the water was changed to Hoagland nutrient solution to provide nutrients to the plants. Hoagland nutrient solution (Hopebiol, China) is prepared according to manufacturer instructions. Briefly, 1.26 g of this product and 0.945 g of $Ca(NO_3)_2$ were dissolved in 1000 mL of deionized water and autoclaved at 115°C for 30 minutes.

Photosynthesis measurement

Photosynthetic efficiency at 30 days was measured. Ten points on each leaf were measured. Pn, Gs, Ci, and Tr were measured using an open gas exchange system (LI-COR Biosciences, Lincoln, NE). Measurement was performed between 08:00-11:00 in the morning. The photosynthetic active radiation was set as 1000 μmol/m^2/s and CO_2 molar fraction was 400 μmol/mol. The instrument was recalibrated after measurement of every 5 samples to obtain a stable measurement.

Soluble sugar and starch contents measurement

For soluble sugar, 80% ethanol was added to fresh samples that were submerged in an 80 ℃ water bath for 30 min. After cooling down to room temperature, samples were centrifuged at 4000 rpm for 10 min. The supernatant was mixed with 2% anthrone and 98% sulfuric acid. The samples were boiled for 10 min, then cooled in ice water to room temperature. Absorbance at 620 nm of the solutions were measured on a microplate reader (Power Wave XS2, BioTek, USA). Glucose solutions with known concentrations were used as standards to calculate the sugar concentration in the samples.

For starch, the precipitates collected from after extraction of the total soluble sugars was resuspended in deionized water and incubated in a 95℃-water bath for 15 min followed by the addition of 52% perchloric acid. The mixture was immediately centrifuged at 4000 rpm for 10 min. The supernatant was diluted 10 times with deionized water and then processed following the same procedure as that for soluble sugar. Absorbance at 620 nm of the solutions were measured on a microplate reader (Power Wave XS2, BioTek, USA). Starch contents were quantified using glucose as the standard with a factor of 0.9 applied for the conversion of glucose to starch.

Enzymes involved in nitrogen fixation and assimilation.

For GS, fresh samples were homogenized in the extraction medium (100 mmol/L hydroxylamine hydrochloride buffer, 1 mmol/L EDTA, 1 mmol/L $MgCl_2$, 10 mmol/L 2-mercaptoethanol) and incubated for 15 minutes at 37°C. Subsequently, the termination solution (0.37 mM $FeCl_3$, 0.2 M trichloroacetic acid and 0.6 mM HCl) was added to the homogenates. The solutions were centrifuged at 4000 g for 10 min and the absorbance of the supernatants was measured at 540 nm. The concentration was calculated based on the calibration curve established with γ-glutamylhydroxamate standard solutions. GS activities were expressed as produce of micromoles of γ-glutamylhydroxamate per minute per milligram protein.

For GOGAT, samples were homogenized in an extraction solution 1 containing of 0.2 mol/L Na_3PO_4, 2 mmol/L EDTA, 50 mmol/L KCl, 1% 2-mercaptoethanol and 0.5% Triton X-100. The mixture was filtered and centrifuged at 2000 g for 10 minutes, then ammonium sulfate was added to the supernatant and precipitate formed. The precipitate was dissolved in the extraction solution again and the procedure was repeated 3 times. The obtained solution was passed through Sephadex G-75 column to obtain the enzyme solution. Solution 2 (10 mM α-ketoglutarate, 1 mM KCl, 37.5 mM Tris-HCl (pH 7.6), 0.6 mM NADH and 8 mM glutamine) was then added to the enzyme solution, and the absorbance at 340 nm was measured at 20 secs and 5 mins

and 20 secs. GOGAT activities were expressed as a reduction of micromoles of NADH per minute per milligram protein.

For GDH, fresh samples were added to the solution containing 0.1 M Tris-HCl (pH 8.3), 1.5 M NH_4Cl, 0.225 M α-Ketoglutaric acid and 7.5 mM NADH, and allowed extraction for 2 minutes. The absorbance at 340nm was measured using a microplate reader (Power Wave XS2, BioTek, USA). GDH activities were expressed as a reduction of micromoles of NADH per minute per milligram protein.

For AO and XDH, samples were homogenized in extraction solution (0.25 mM Tris–HCl buffer, pH 8.5), 1 mM ethylene diamine tetra acetic acid (EDTA), 1 mM dithiothreitol (DTT), 3 mM reduced glutathione (GSH), 3% polyvinylpolypyrrolidone (PVPP)). After 10 min, the mixture was centrifuged at 12,000 rpm and 4 °C for 20 min. A solution (50 mM potassium phosphate (pH 7.4), 0.002% DCIP, 0.1 mM PMS, and 2 mM indole-3-aldehyde) was then added to measure the AO activities. For XDH, another solution (50 mM Tris-HCl (pH 8.48), 0.002% DCIP, 0.1 mM PMS, and 0.6 mM hypoxanthine) was added instead. For both AO and XDH, the absorbance at 600 nm was measured. AO and XDH activities were expressed as a reduction of micromoles of DCIP per minute per gram of fresh sample.

For NR, the samples were homogenized with the solution (GST-fusion protein, 0.1 mM potassium phosphate buffer (pH 7.5), 0.1 mM KNO_3 and 0.25 mM nicotinamide adenine dinucleotide phosphate (NADPH)). The homogenates were centrifuged at 3500 rpm and 4 °C for 15 min. The supernatants were spiked with 2M hydrochloric acid, 1% sulphonamide and 0.02% N-(1-naphthyl)-ethylene-ammonium dichloride (NED). After 10 min, the absorbance at 540 nm was measured. NR activities were expressed as produce of micromoles of NO_2^- per minute per gram fresh sample.

According to Buendia-Claveria et al. (1986)[3], acetylene reduction method was used to determine the activity of nitrogenase. In brief, the roots of the soybean were cut and washed and blotted dry with absorbent paper. The roots were sealed in a 100 mL conic flask. Ten mL of air was withdrawn from the reaction flask using vacuum syringe, and 10 ml of acetylene gas was injected as the reaction gas. The reaction was

maintained at 28℃ thermostat for 3h. Ethylene concentration in the solution was measured by gas chromatography (Aligent 7820A, Agilent Technologies, USA). The parameter for the measurement was as follows: injector temperature, 100 °C; column temperature, 70℃; Detector temperature, 250℃; H_2, 40 mL/min; air, 400 mL/min; N_2, 5 mL/min.

Metabolomics analysis

The metabolic profile of leaf samples collected at 30 days were analyzed by a gas chromatograph-mass spectrometer (Trace 1310/TSQ 9000, Thermo, USA), with a DB-5MS fused-silica capillary column (Agilent, USA). The carrier gas was high-purity helium, the flow rate was 1.2 ml/min and the temperature of the injector was 300 °C. Electron bombardment ion source (EI) temperature was 330 °C, and transmission line temperature was 280 °C. The scanning mode was Full Scan for reaction detection, and the quality scanning range was m/z 50-500.

All peak signal intensities were segmented and normalized, and then de-redundancy and peak merging were performed to obtain the data matrix. Student's T-test and Fold change analysis were used to compare the metabolites between the two groups. The screening criteria were the VIP value of the first principal component in the OPLS-DA model, >1, and the p-value value of the T-test <0.05. KEGG (https://www.kegg.jp/) was used for the enrichment analysis of metabolic pathways.

Optical microscope and confocal laser scanning microscope observation

Nodules with 500 mg/kg MoS_2 NPs, 500 mg/kg MoS_2 NS, 500 mg/kg Na_2MoO_4, and control groups were observed by light microscopy. The samples were fixed in formaldehyde-acetic acid-ethanol (FAA) fixative for 1 h at room temperature. Then, the samples were dehydrated in different concentrations of ethanol (30%, 50%, 70%,

80%, 90%, 95%, 100%) for 60 min. The dehydrated samples were embedded in paraffin wax. The sections were stained using the toluidine blue staining technique and observed using a light microscope (Zeiss-AX10).

Fresh nodules were embedded and sectioned with a vibrating microtome (Leica, Wetzlar, Germany) at a section thickness of 70 μm. Sections were incubated with 5 mM ROS stain for 60 min protected from light and then excess probe was rinsed with PBS. The sections were covered with a slide after dropping the sealer. Confocal laser scanning microscope (Olympus, Japan) was used to observe and acquire images (CY3 excitation wavelength 510-560 nm, emission wavelength 590 nm, red light emission).

Collection of root exudates, soil leachate and soil pore water

Root exudates and soil leachate were collected using the methods described by Cervantes-Aviles et al[4]. For root exudates, the 30-day-old soybean was placed in 100 ml of 0.1 mM $CaCl_2$ solution for 12 hours to collect the root exudates. After 12 hours, 0.1 mM $CaCl_2$ solution containing the root exudate was filtered through a syringe filter (0.45 μm) to remove particulate matter.

The soil used to grow soybeans in this experiment was used to extract soluble compounds. Deionized water was added to the soil at a ratio of 4:1 (v/v) and the mixture was shaken at 200 rap/min for 12 hours on an incubator shaker (Kebaite, China). Then, the mixture was centrifuged at 4000 rpm for 10 min. The supernatant was collected and used for the dissolution experiment.

Soil pore water was collected using soil pore water sampler (Eijkelkamp Agrisearch Equipment, The Netherlands) coupled with a 5 ml vacuum bottle. With plant soils were used. Soil pore water was collected at days 15, 30, 45, 60, 75, and 90 of incubation, respectively.

Pot Experiment on Drought and Heat Stress

The soils were the same as those used in the greenhouse experiments. MoS_2 NPs were

mixed with the soil thoroughly in plastic pots to achieve final concentrations of 10 mg/kg; untreated soil was used as a control. The methods of soybean seed germination and rhizobia inoculation were the same as in the greenhouse experiment. Soybean seedlings (5 days old) of uniform size were transferred into the pots. Seedlings were grown in incubator 1 with 70% relative humidity, 20,000 lx light intensity, 14 h 28°C/8 h 24°C (day/night) for 15 days and soil moisture content maintained at 70%. Then drought stress and heat stress treatments were performed. The heat stress treatment was soybean was replaced to incubator 2 with 70% relative humidity, 20,000 lx light intensity, 14 h 35°C/8 h 30°C (day and night) and soil moisture content maintained at 70%. The drought stress treatment was continued in incubator 1 and soil moisture content maintained at 35%. The no-stress treatment continued to grow in incubator 1 and soil moisture content maintained at 70%. The plants were harvested at 10 d for analyses.

Section 2. Supplementary data, results and discussion.

Note: Data are presented as Figures and Tables followed by results and discussion.

Human health risk evaluation of soybean grain

The development of new nano-fertilizers should first consider their safety, and in this study, human health risk was assessed for Mo content in soybean grains. The target hazard factor (THQ) was used to assess the potential non-carcinogenic health risk of soybean grains, which was considered to be potentially non-carcinogenic when THQ > 1 and vice versa. The predicted daily intake (EDI) was the daily Mo intake from consumption of soybean grains, and a health risk was considered when the EDI of Mo in food exceeded 5 μg/kg/bw/day. As shown in **Supplementary Table 1**, the EDI of Mo from soybean grains treated with three concentrations of MoS_2 Bulk was below the reference value with THQ < 1 and no potential non-carcinogenic health risk. The EDI of Mo in soybean grains treated with three concentrations of MoS_2 NS

and Na_2MoO_4 was higher than the reference value and THQ > 1, with potential non-carcinogenic health risk. 10 and 100 mg/kg MoS_2 NPs-treated soybean grains had EDI values lower than the reference value (5 μg/kg/bw/day) for both males and females, THQ < 1, and 500 mg/kg The EDI of MoS_2 NPs treated soybean grains was higher than the reference value with THQ > 1. Therefore, 10 and 100 mg/kg of MoS_2 NPs treated soybean grains have no health risk to humans and are safe as nano-molybdenum fertilizer to enhance soybean yield.

Effects of Mo fertilizers on the soybean growth, photosynthesis, C/N accumulation and metabolic profiles (30 days data)

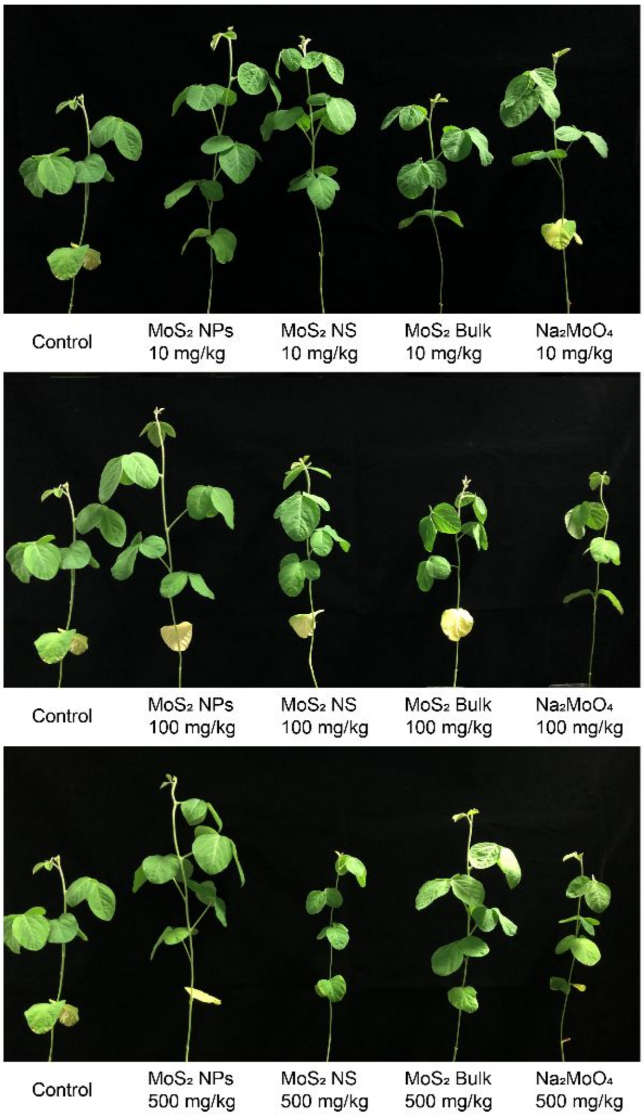

Supplementary Figure 1. Phenotypic images of soybean seedlings (30 d).

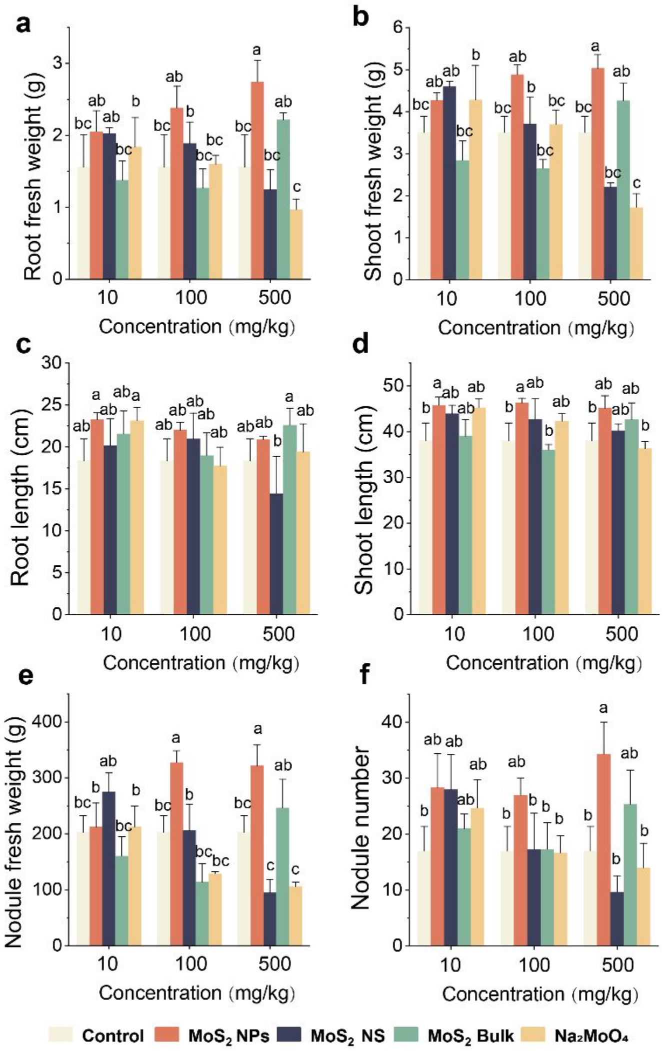

Supplementary Figure 2. The phenotype of soybean after treatment with Mo fertilizer for 30 days, including the fresh weight of roots (a) and shoots (b), the length of roots (c) and shoots (d) and the fresh weight (e) and number (f) of nodules. The data are shown as the mean ± SD (n= 6). Statistical significance was tested with one-way ANOVA analysis with a Tukey's test. Different lowercase letters indicate significant difference between groups at $P< 0.05$.

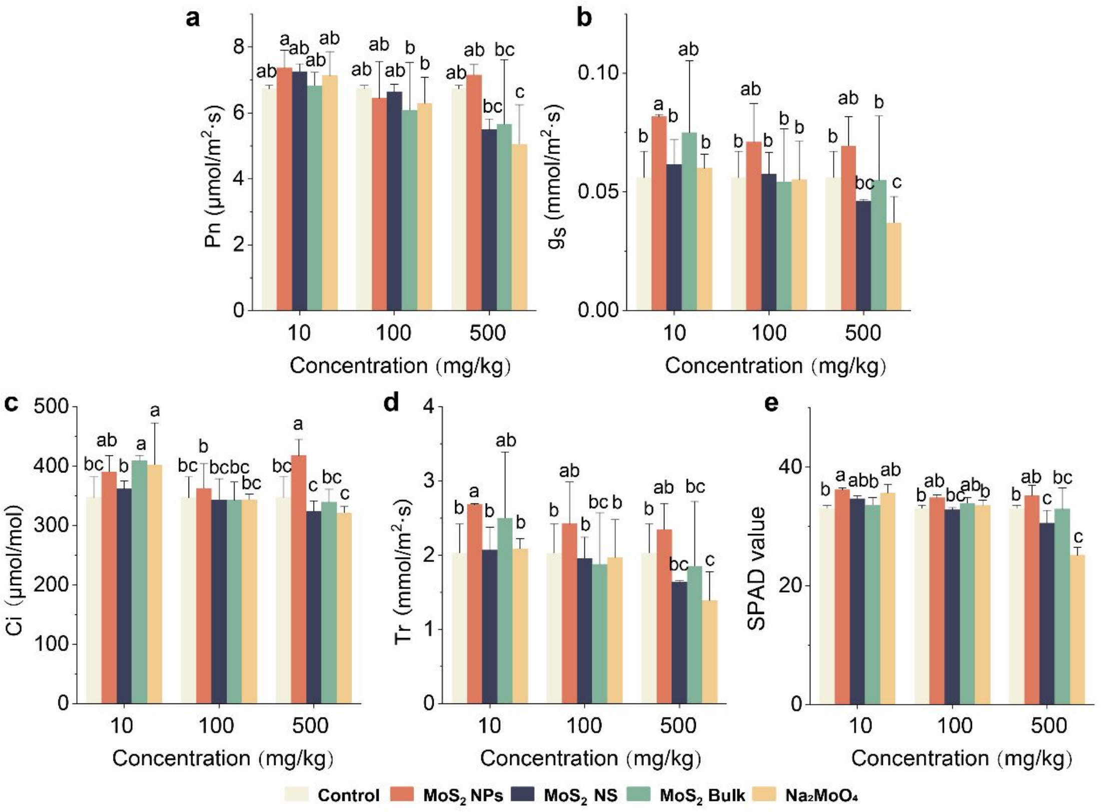

Supplementary Figure 3. Photosynthetic parameters of soybean after treatment with Mo fertilizer for 30 days, including Pn (a) gs (b), Ci (c), Tr (d) and SPAD value (e). The data are shown as the mean ± SD (n= 6). Statistical significance was tested with one-way ANOVA analysis with a Tukey's test. Different lowercase letters indicate significant difference between groups at $P< 0.05$.

The degree of nutrient availability at each stage is critical for soybean growth. Therefore, we first investigated whether the promoting effect of MoS_2 NPs also occurs at the early growth stage of soybean, which is a crucial stage for nodule formation and the basis for late growth and productivity of soybean. Soybean seedlings were harvested at 30 days (V6) and their phenotypes were measured. The results illustrated that MoS_2 NPs promoted soybean growth in comparison to MoS_2 NS, MoS_2 Bulk and Na_2MoO_4, with no negative biological effects on plants in a wide range of concentrations (10, 100 and 500 mg/kg). Plant phenotype is the most intuitive manifestation of the biological effects of nanoparticles. The plant phenotype

data were shown in **Supplementary Fig. 1**. The biomass of soybean treated with MoS_2 NPs (10, 100 and 500 mg/kg) was increased by 32%, 53% and 76% in roots and 22%, 39% and 44% in shoots, compared with control. MoS_2 NS and Na_2MoO_4 at 500 mg/kg significantly reduced shoot biomass by 37% and 51% (Supplementary Fig. 2). As the growth of soybean is inextricably linked to the nitrogen fixation of the nodules and as the molybdenum cofactor (Fe-Moco), which consists of molybdenum, is the active center of the nitrogenase[5, 6], we compared the effects of four materials on nodule growth. As we expected, the growth of nodules shared a similar trend as the biomass of the plant. MoS_2 NPs (100 and 500 mg/kg) significantly increased nodule biomass by 61% and 59%. The nodule biomass was decreased by MoS_2 NS and Na_2MoO_4 at 500 mg/kg, while increased by MoS_2 Bulk only at 500 mg/kg. Corresponding to the phenotypic characteristics, photosynthesis was promoted by 10 mg/kg of MoS_2 NPs and inhibited by 500 mg/kg of MoS_2 NS and Na_2MoO_4. Accordingly, 500 mg/kg MoS_2 NS and Na_2MoO_4 also significantly decreased photosynthetic parameters. 500 mg/kg Na_2MoO_4 significantly decreased photosynthetic rate (P_N), transpiration rate (Tr) and stomatal conductance (Gs) by 25%, 31% and 34%, respectively. Conversely, 10 mg/kg MoS_2 NPs increased transpiration rate (Tr) and stomatal conductance (Gs) by 32% and 46% and 500 mg/kg MoS_2 NPs increased intercellular CO_2 concentration (Ci) by 20% (**Supplementary Fig. 3a-d**). The SPAD value was increased by 10 mg/kg MoS_2 NPs by 9% and decreased by 500 mg/kg MoS_2 NS and Na_2MoO_4 by 8% and 24% (**Supplementary Fig. 3e**). The improvement of Ci will increase plant biomass and yield[7, 8]. Stomata opening is essential for the diffusion of CO_2 into the leaves[9]. Therefore, the MoS_2 NPs facilitated gas exchange may have contributed to improved seedling biomass and yield at maturity. Thus, our results suggest that MoS_2 NPs started to stimulate plant growth at the seedling stage, especially by increasing nodule formation, and that this effect persists until maturity.

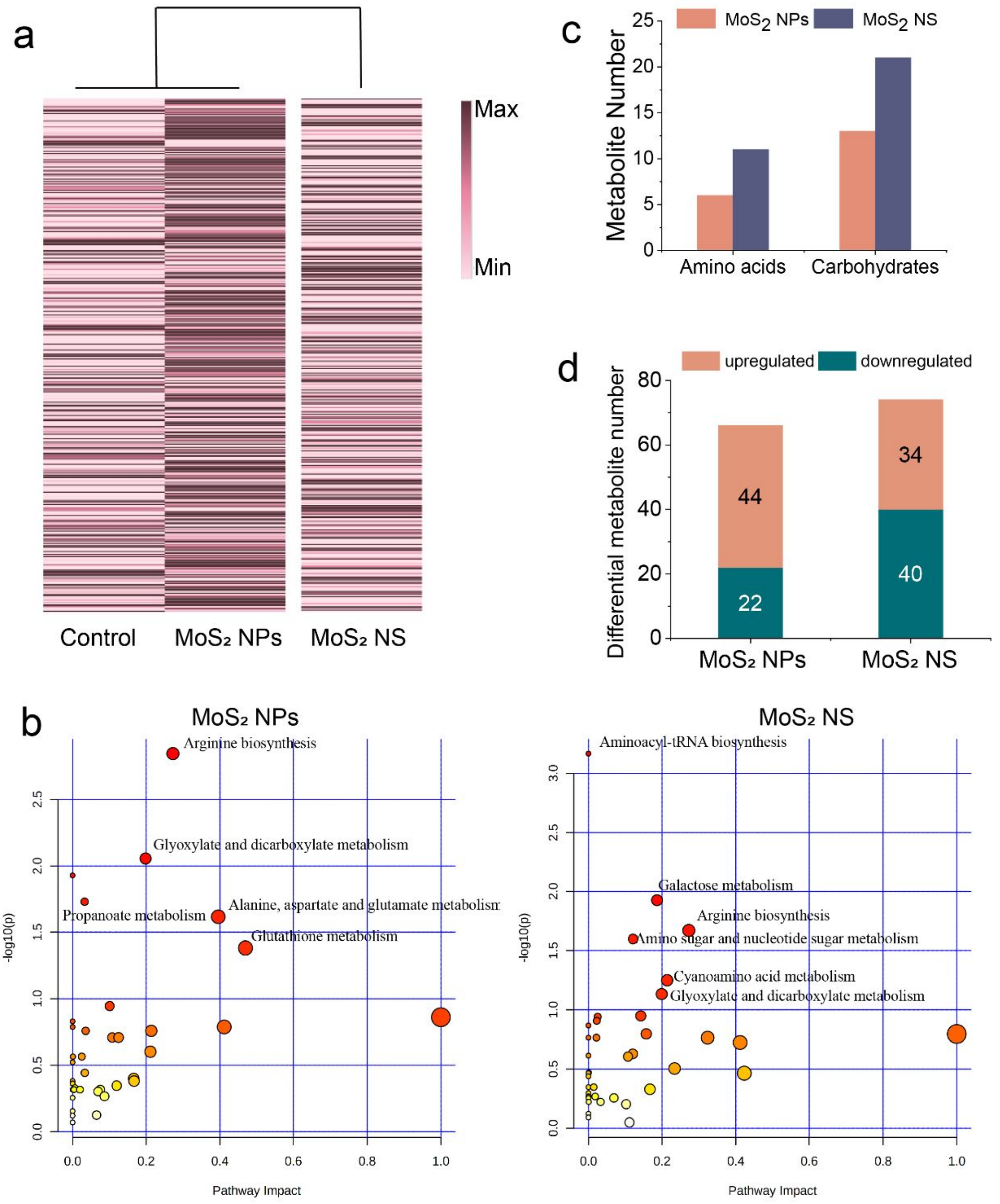

Supplementary Figure 4. Metabolomics of shoots after 30 days of treatments with 500 mg/kg nanostructures (MoS_2 NPs and MoS_2 NS) and control groups. Cluster analysis of the union of metabolites (a); the number of differential metabolites involved in different biology (b); the number of differential metabolites treated by MoS_2 NPs and MoS_2 NS (c).

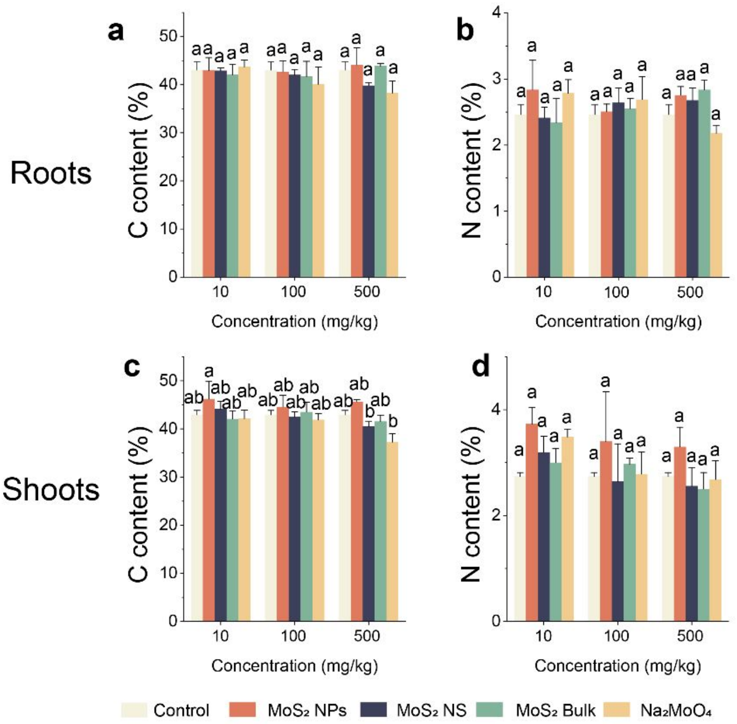

Supplementary Figure 5. Carbon and nitrogen accumulation in plant roots (a, b) and shoots (c, d) after 30 days of treatments with Mo fertilizers. The data are shown as the mean ± SD (n= 6). Statistical significance was tested with one-way ANOVA analysis with a Tukey's test. Different lowercase letters indicate significant difference between groups at $P< 0.05$.

Effects of Nano-MoS_2 on the soil microbes

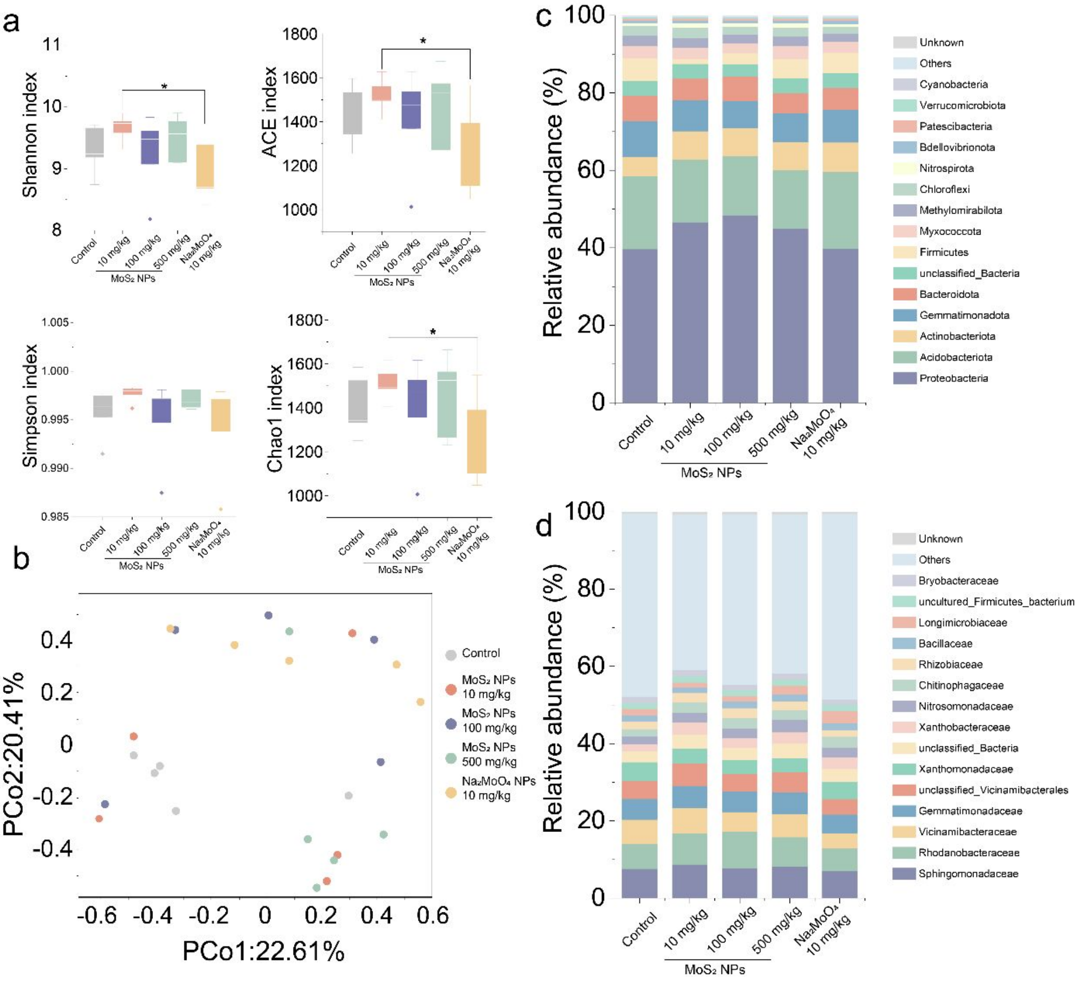

Supplementary Figure 6. Effects of materials on microbial community diversity in soil. **a.** Alpha diversity analysis. Chao1 index and Ace index indicate species richness and Shannon index and Simpson index indicate species diversity. **b**, PCoA plot depicts the Bray-Curtis distance of bacterial communities. **c**, Dominate bacterial communities of the different treatments at the phylum level. **d**, Dominate bacterial communities of the different treatments at the family level.

The Shannon, ACE, and Chao1 index results suggested that the microbial species diversity and richness were higher in soil treated with 10 mg/kg MoS_2 NPs than 10 mg/kg (**Supplementary Figure 6a**). PCoA analysis plots (β-diversity) explained the same conclusion, with no significant difference in soil microbial diversity between the treatment group (10, 100 and 500 mg/kg MoS_2 NPs) and the control group (**Supplementary Figure 6b**).

The top ten dominant bacterial phyla with the highest relative abundance were Proteobacteria, Acidobacteria, Actinobacteria, Gemmatimonadetes, bacteroidota, which accounted for about 80% of all sequence reads (**Supplementary Figure 6c**). Among them, the relative abundance of proteobacteria was highest in all the treatments, ranging from 39.7% (the control) to 48.3% (100 mg/kg MoS_2 NPs treatment). Compared to the control, 10, 100 and 500 mg/kg MoS_2 NPs treatments increased the relative abundance of Proteobacteria by 17%, 22% and 13%, respectively. Proteobacteria plays an vital role in the global C, N, and P cycle[10]. Proteobacteria are the predominant bacterial phylum in soils, which contain a large number of nitrogen-fixing bacteria[11]. Thus, the increased abundance of Proteobacteria represented an increase in the relative abundance of rhizobia and other nitrogen-fixing bacteria, which explained the accumulation of nitrogen in MoS_2 NPs-treated soybean. Compared to the control, 10, 100 and 500 mg/kg MoS_2 NPs treatments reduced the relative abundance of Acidobacteria by 13%, 18% and 19%, respectively. Soil pH is a key factor that shapes microbial community composition, with Acidobacteria being a common occurrence in particularly acidic soil environments[12]. Therefore, MoS_2 NPs treatment probably influenced soil pH resulting in reduced abundance of Acidobacteria. Compared to the control, 10, 100 and 500 mg/kg MoS_2 NPs treatments increased the relative abundance of Actinobacteriota by 45%, 43% and 42%, respectively. Soil pH is a crucial determinant of the abundance of Actinobacteriota, as these microorganisms thrive more effectively in high-pH soil[13]. This suggests that MoS_2 NPs probably increased the abundance of actinomycetes by regulating soil pH. In addition, Actinobacteriota can enhance the resistance of plants to biological stresses, which reduces the input of chemical pesticides[14].

From family level, 10, 100 and 500 mg/kg MoS_2 NPs treatment increased the abundance of Rhodanobacteraceae (25%, 50% and 19%), Rhizobiaceae (29%, 34% and 17%), respectively (**Supplementary Figure 6d**). Rhizobiaceae belongs to rhizobiales that have biological nitrogen fixation ability[15]. Rhodanobacteraceae is considered to be organic matter decomposers and/or plant growth-promoting bacteria[16]. MoS_2 NPs also increased the level of Nitrosomonadaceae, which indicates an increase in ammonia in the soil[17]. It is possible that the application of MoS_2 NPs increased the level of rhizobiales, which increased the nitrogen fixation capacity of the soil, thus increasing ammonia content in the soil. In addition, MoS_2 NPs enhanced Chitinophagaceae level, which could enhance crop resistance to soil pests and

diseases[18]. In summary, MoS_2 NPs treatment promoted soil N cycling and enhanced resistance to pests and diseases.

Nano-MoS_2 as an enhancer of plant tolerance to abiotic stress

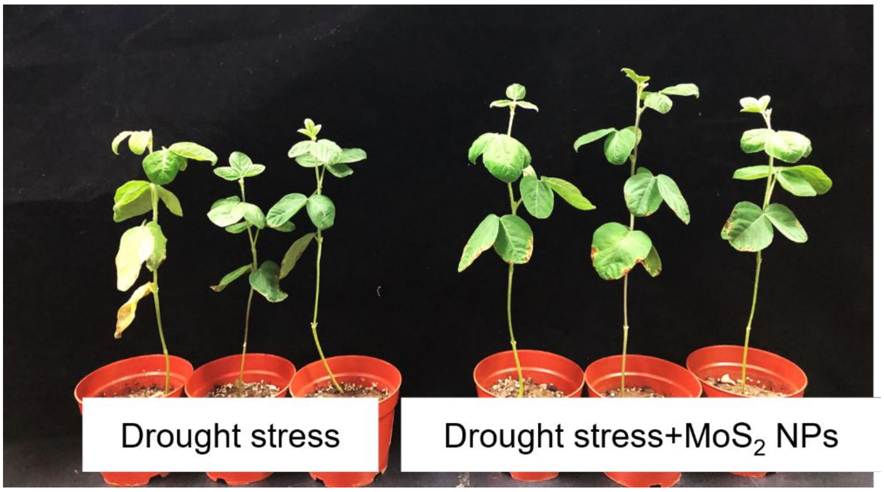

Supplementary Figure 7. Phenotypic images of soybean seedlings under drought stress.

To investigate the protective strategies of MoS_2 NPs against abiotic stresses in plants, MoS_2 NPs were applied to plants subjected to heat and drought stress. Drought stress, high temperature stress, drought stress + 100 mg/kg MoS_2 NPs treatment, and high temperature stress + 100 mg/kg MoS_2 NPs treatment were set. Soybean was treated with stress after one week of growth and the stress duration was 10 days. The results showed that the biomass of shoots and nodules of plants under drought stress decreased by 0.37 and 0.39 times, respectively, compared with the control (**Fig. 7a**). MoS_2 NPs treatment increased the shoot and nodule biomass by 0.25 and 0.37 times, respectively, compared to soybean exposed to drought stress (**Fig. 7a**). There was no significant difference between the MoS_2 NPs treatment and the control. Compared to the control, nodule and shoot biomass of soybean exposed to heat stress were reduced by 0.38 and 0.43 times, respectively. Compared to soybean exposed to heat stress, MoS_2 NPs increased the nodule biomass of the soybean by 0.19 times and did not contribute noticeably to the reduction in maintaining root and shoot biomass. The

results illustrated that MoS_2 NPs maintained the reduction in biomass, successfully mitigated drought stress, and mitigated the reduction in nodule biomass under heat stress (**Fig. 7b**).

Compared to the control, net photosynthetic efficiency (Pn), stomatal conductance (Gs) and intercellular CO_2 concentration (Ci) of soybean exposed to drought stress were significantly reduced by 0.23, 0.56 and 0.25 times (**Fig. 7b**). Compared to soybean exposed to drought stress, MoS_2 NPs treatment increased Pn, Gs and Ci of plants by 0.1, 0.56 and 0.25 times, which indicated that photosynthesis was inhibited. Pn and Ci of soybean treated with MoS_2 NPs were not significantly different from the control. Pn, Gs and Ci of soybean exposed to heat stress were significantly decreased by 0.27, 0.41 and 0.41 compared to the control. MoS_2 NPs treatment increased the Gs and Ci of soybean by 0.13 and 0.47 times compared to soybean exposed to heat stress (**Fig. 7b**). The Ci of MoS_2 NPs-treated soybean was not significantly different from the control. MoS_2 NPs alleviated the inhibition of photosynthesis by drought stress and increased intercellular CO_2 concentration under heat stress. The reduction of crop yield due to drought stress is related to the decrease of photosynthetic efficiency, which is mainly attributed to the inhibition of photosynthesis by drought stress through stomatal reduction of CO_2 uptake[19]. The results of short-term experiments showed that MoS_2 NPs promoted leaf gas exchange through accelerated nitrogen assimilation. Therefore, the inhibition of photosynthesis under drought stress alleviated by MoS_2 NPs may be related to the promotion of nitrogen assimilation by MoS_2 NPs.

Abiotic stresses usually lead to lipid peroxidation and reduced cell membrane fluidity, and MDA can directly respond to the degree of membrane damage. Compared with the control, drought stress increased the MDA content 3.45 and 0.91 times in roots and shoots, and significantly increased the CAT, POD and SOD activities in roots and shoots. Compared to plants exposed d to drought stress, MoS_2 NPs treatment reduced the MDA content in soybean roots and shoots by 0.14 and 0.26 times, respectively. MoS_2 NPs decreased the activity of CAT, SOD in shoots, elevated

the activity of POD in shoots, and decreased the activity of POD in roots. The activities of CAT and SOD in MoS_2 NPs-treated soybean shoots were not significantly different from the control (**Fig. 7c**).

Compared to the control, heat stress elevated MDA content by 1.96 and 1.94 times in roots and shoots, respectively, increased POD and SOD activities in shoots, and SOD activities in roots. Compared to the stressed plants, the MoS_2 NPs treatment slightly reduced the MDA content of roots and shoots by 0.03 and 0.05 times, respectively. The activity of POD was not significantly higher in MoS_2 NPs-treated soybean shoots compared to the control. Thus, MoS_2 NPs treatment alleviated the oxidative damage triggered by drought stress, but did not significantly reduce the oxidative damage triggered by heat stress (**Fig. 7c**).

Section 3. Supplementary Table

Table S1

Health risk assessment of soybean grains

Treatments		Estimated daily intake (EDI) (μg)		Target hazard factor (THQ)	
Materials	Concentration (mg/kg)	Male	Female	Male	Female
MoS_2 NPs	10	0.66	0.85	0.13	0.17
	100	3.79	4.85	0.76	0.97
	500	14.88	19.03	2.98	3.81
MoS_2 NS	10	7.78	9.95	1.56	1.99
	100	27.45	35.10	5.49	7.02
	500	85.70	109.58	17.14	21.92
MoS_2 Bulk	10	0.52	0.66	0.10	0.13
	100	1.15	1.47	0.23	0.29
	500	2.63	3.37	0.53	0.67
Na_2MoO_4	10	10.06	12.87	2.01	2.57
	100	52.26	66.81	10.45	13.36
	500	122.17	156.20	24.43	31.24

Table S2

Current approaches to improve the efficiency of soybean production

Approaches	Treatments	Efficiency outcomes of yields （normal condition）	Refs
Gene editing	GmSWEET10a/b	11-20%	20
	POWR1	4.7%	21
	GmST05	12%	22
	GmMYB14	15.9%	23
	Ln locus	8-10%	24
	hybrid	10%-20%	25
	Gibberellin-3-beta-dopamine	~12%	26
	GmJAGGED1	8.81%	27
	SOC1	13.5-23.2%	28
	CIF-6-1	<30%	29
	GmPT7	15-26%。	30
	Arabidopsis thaliana BBX32	<10%	31
	sHSP26	14.5	32
Beneficial microorganism	Bacillus subtilis	4%	33
	Microbial inoculant	3%	34
	R. cellulosilyticum strain	14%	35
	Biomaphos	9%	13
	phosphorus-solubilizing bacteria +vesicular arbuscular microorganisms	4.1%	36
	phosphorus-solubilizing bacteria + arbuscular mycorrhiza	3.4%	37

arbuscular mycorrhiza	14.5%	38

Table S3

BET surface area of the materials.

Materials	BET surface area (m^2/g)
MoS_2 NPs	9.2883
MoS_2 NS	17.8713
MoS_2 Bulk	7.7801

Table S4

Soil parameters used in the experiment.

soil mechanical composition	pH	Electrical conductivity (μS/cm)	organic matter (g/kg)	N (g/kg)	P (mg/kg)	K (mg/kg)	S (mg/kg)	Mo (mg/kg)
silt 14%, sand 84%, clay 4%	6.76	801	3.87 %	1.57	49.6	131.2	350.23	1.20

Table S5

Limit of detection, precision and recovery data of ICP-MS for the selected elements.

Elements	Limits of Detection (μg/L)	Recovery	Correlation coefficient (R^2)	RSD
Mo	0.017	98.1%	0.9999	1.7
K	0.043	102.8%	0.9998	2.5
Ca	0.048	98.5%	0.9998	2.5
Mg	0.033	101.8%	0.9999	2.1
P	0.036	98.0%	0.9999	1.9
Fe	0.042	101.3%	0.9997	2.6
Mn	0.029	101.7%	0.9996	2.3
Cu	0.064	97.3%	0.9997	1.8
Zn	0.048	97.1%	0.9998	2.6

Table S6

Primer sequences used for RT-PCR analysis

Gene	Primer	Sequence
CNX1	Forward	5’-AGTGAACGATGCCTCCGATG-3’
CNX1	Reverse	5’-CCACGATTCAGATGCCCAGT-3’
CNX2	Forward	5’-CTCTGGTACCGGCGAAGTT-3’
CNX2	Reverse	5’-CACGCGTCAACTCAACAAAGT-3’
CNX6	Forward	5’-CACAACTCGCGACACCTTTG-3’
CNX6	Reverse	5’-CTACAAGCCTCCAGCGCAT-3’
XDH	Forward	5’-CGGAGTTCGCAGATTGCTGT-3’
XDH	Reverse	5’-GCTTCCCAATCCCTCCACTG-3’
AO	Forward	5’-GTTGTGGCGCTTGTGTAGTT-3’
AO	Reverse	5’-CAGGTGGCTCTGGACAAGTAG-3’

NR	Forward	5’-AATTCCCTCACCGCGAGTTT -3’
NR	Reverse	5’-CATACTTGGACCCACCACCC-3’
Actin11	Forward	5’-CGGTGGTTCTATCTTGGCATC -3’
Actin11	Reverse	5’-GTCTTTCGCTCCAATAACCCTA-3’

References:

1. Wang, Z. Y.; von dem Bussche, A.; Qiu, Y.; Valentin, T. M.; Gion, K.; Kane, A. B.; Hurt, R. H., Chemical dissolution pathways of MoS2 nanosheets in biological and environmental media. *Environmental Science & Technology* **2016,** *50* (13), 7208-7217.

2. Eda, G.; Yamaguchi, H.; Voiry, D.; Fujita, T.; Chen, M.; Chhowalla, M., Photoluminescence from chemically exfoliated MoS2. *Nano Letters* **2011,** *11* (12), 5111-5116.

3. Buendia-Claveria, A. M.; Ruiz-Sainz, J. E.; Cubo-Sanchez, T.; Perez-Silva, J., Studies of symbiotic plasmids in Rhizobium trifolii and fast-growing bacteria that nodulate soybeans. *Journal of Applied Bacteriology* **1986,** *61* (1), 1-9.

4. Cervantes-Aviles, P.; Huang, X.; Keller, A. A., Dissolution and aggregation of metal oxide nanoparticles in root exudates and soil leachate: Implications for nanoagrochemical application. *Environmental Science & Technology* **2021,** *55* (20), 13443-13451.

5. Cao, X.; Yue, L.; Wang, C.; Luo, X.; Zhang, C.; Zhao, X.; Wu, F.; White, J. C.; Wang, Z.; Xing, B., Foliar application with iron oxide nanomaterials stimulate nitrogen fixation, yield, and nutritional quality of soybean. *Acs Nano* **2022,** *16* (1), 1170-1181.

6. Schwarz, G.; Mendel, R. R.; Ribbe, M. W., Molybdenum cofactors, enzymes and pathways. *Nature* **2009,** *460* (7257), 839-847.

7. Kimball, B. A., Crop responses to elevated CO2 and interactions with H2O, N, and temperature. *Current Opinion in Plant Biology* **2016,** *31*, 36-43.

8. Long, S. P.; Zhu, X. G.; Naidu, S. L.; Ort, D. R., Can improvement in photosynthesis increase crop yields? *Plant Cell and Environment* **2006,** *29* (3), 315-330.

9. Zhang, M.; Wang, Y.; Chen, X.; Xu, F.; Ding, M.; Ye, W.; Kawai, Y.; Toda, Y.; Hayashi, Y.; Suzuki, T.; Zeng, H.; Xiao, L.; Xiao, X.; Xu, J.; Guo, S.; Yan, F.; Shen, Q.; Xu, G.; Kinoshita, T.; Zhu, Y., Plasma membrane H+-ATPase overexpression increases rice yield via simultaneous enhancement of nutrient uptake and photosynthesis. *Nat. Commun.* **2021,** *12* (1), 735.

10. Spain, A. M.; Krumholz, L. R.; Elshahed, M. S., Abundance, composition, diversity and novelty of soil Proteobacteria. *The ISME Journal* **2009,** *3* (8), 992-1000.

11. Delmont, T. O.; Quince, C.; Shaiber, A.; Esen, Ö. C.; Lee, S. T. M.; Rappé, M. S.; McLellan, S. L.; Lücker, S.; Eren, A. M., Nitrogen-fixing populations of Planctomycetes and Proteobacteria are abundant in surface ocean metagenomes. *Nature Microbiology* **2018,** *3* (7), 804-813.

12. Kalam, S.; Basu, A.; Ahmad, I.; Sayyed, R. Z.; El-Enshasy, H. A.; Dailin, D. J.; Suriani, N. L., Recent Understanding of Soil Acidobacteria and Their Ecological Significance: A Critical Review. *Frontiers in Microbiology* **2020,** *11, 580024*.

13. Lupwayi, N. Z.; Blackshaw, R. E.; Geddes, C. M.; Dunn, R.; Petri, R. M., Multi-year and multi-site effects of recurrent glyphosate applications on the wheat rhizosphere microbiome. *Environmental Research* **2022,** *215*, 114363.

14. Xu, Z.-M.; Zhang, Y.-X.; Wang, L.; Liu, C.-G.; Sun, W.-M.; Wang, Y.-F.; Long, S.-X.; He, X.-T.; Lin, Z.; Liang, J.-L.; Zhang, J.-X., Rhizobacteria communities reshaped by red mud based passivators is vital for reducing soil Cd accumulation in edible amaranth. *Science*

of The Total Environment **2022,** *826*, 154002.

15. Hakoyama, T.; Niimi, K.; Watanabe, H.; Tabata, R.; Matsubara, J.; Sato, S.; Nakamura, Y.; Tabata, S.; Jichun, L.; Matsumoto, T.; Tatsumi, K.; Nomura, M.; Tajima, S.; Ishizaka, M.; Yano, K.; Imaizumi-Anraku, H.; Kawaguchi, M.; Kouchi, H.; Suganuma, N., Host plant genome overcomes the lack of a bacterial gene for symbiotic nitrogen fixation. *Nature* **2009,** *462* (7272), 514-517.

16. Shen, F.-T.; Lin, S.-H., Priming Effects of Cover Cropping on Bacterial Community in a Tea Plantation. *Sustainability* **2021,** *13* (8), 4345.

17. Clark, I. M.; Hughes, D. J.; Fu, Q.; Abadie, M.; Hirsch, P. R. J. S. R., Metagenomic approaches reveal differences in genetic diversity and relative abundance of nitrifying bacteria and archaea in contrasting soils. **2021,** *11* (1), 1-9.

18. Carrion, V. J.; Perez-Jaramillo, J.; Cordovez, V.; Tracanna, V.; de Hollander, M.; Ruiz-Buck, D.; Mendes, L. W.; van Ijcken, W. F. J.; Gomez-Exposito, R.; Elsayed, S. S.; Mohanraju, P.; Arifah, A.; van der Oost, J.; Paulson, J. N.; Mendes, R.; van Wezel, G. P.; Medema, M. H.; Raaijmakers, J. M., Pathogen-induced activation of disease-suppressive functions in the endophytic root microbiome. *Science* **2019,** *366* (6465), 606-+.

19. Bose, J.; Munns, R.; Shabala, S.; Gilliham, M.; Pogson, B.; Tyerman, S. D., Chloroplast function and ion regulation in plants growing on saline soils: lessons from halophytes. *Journal of Experimental Botany* **2017,** *68* (12), 3129-3143.

20. Wang, S.; Liu, S.; Wang, J.; Yokosho, K.; Zhou, B.; Yu, Y.-C.; Liu, Z.; Frommer, W. B.; Ma, J. F.; Chen, L.-Q.; Guan, Y.; Shou, H.; Tian, Z., Simultaneous changes in seed size, oil content and protein content driven by selection of SWEET homologues during soybean

domestication. *National Science Review* **2020,** *7* (11), 1776-1786.

21. Goettel, W.; Zhang, H.; Li, Y.; Qiao, Z.; Jiang, H.; Hou, D.; Song, Q.; Pantalone, V. R.; Song, B.-H.; Yu, D.; An, Y.-q. C., POWR1 is a domestication gene pleiotropically regulating seed quality and yield in soybean. *Nat. Commun.* **2022,** *13* (1), 3051.

22. Duan, Z.; Zhang, M.; Zhang, Z.; Liang, S.; Fan, L.; Yang, X.; Yuan, Y.; Pan, Y.; Zhou, G.; Liu, S.; Tian, Z., Natural allelic variation of GmST05 controlling seed size and quality in soybean. *Plant Biotechnology Journal* **2022,** *20* (9), 1807-1818.

23. Chen, L.; Yang, H.; Fang, Y.; Guo, W.; Chen, H.; Zhang, X.; Dai, W.; Chen, S.; Hao, Q.; Yuan, S.; Zhang, C.; Huang, Y.; Shan, Z.; Yang, Z.; Qiu, D.; Liu, X.; Tran, L.-S. P.; Zhou, X.; Cao, D., Overexpression of GmMYB14 improves high-density yield and drought tolerance of soybean through regulating plant architecture mediated by the brassinosteroid pathway. *Plant Biotechnology Journal* **2021,** *19* (4), 702-716.

24. Liu, S.; Zhang, M.; Feng, F.; Tian, Z., Toward a “Green Revolution” for Soybean. *Molecular Plant* **2020,** *13* (5), 688-697.

25. Palmer, R. G.; Gai, J.; Sun, H.; Burton, J. W., Production and evaluation of hybrid soybean. In *Plant Breeding Reviews*, 2001; Vol. 21, pp 263-307.

26. Hu, D. Z.; Li, X.; Yang, Z. Y.; Liu, S. L.; Hao, D. R.; Chao, M. N.; Zhang, J. Y.; Yang, H.; Su, X. Y.; Jiang, M. Y.; Lu, S. Q.; Zhang, D.; Wang, L.; Kan, G. Z.; Wang, H.; Cheng, H.; Wang, J.; Huang, F.; Tian, Z. X.; Yu, D. Y., Downregulation of a gibberellin 3 beta-hydroxylase enhances photosynthesis and increases seed yield in soybean. *New Phytologist* **2022,** *235* (2), 502-517.

27. Cai, Z.; Xian, P.; Cheng, Y.; Ma, Q.; Lian, T.; Nian, H.; Ge, L., CRISPR/Cas9-

mediated gene editing of GmJAGGED1 increased yield in the low-latitude soybean variety Huachun 6. *Plant Biotechnology Journal* **2021,** *19* (10), 1898-1900.

28. Han, X.; Wang, D.; Song, G.-q., Expression of a maize SOC1 gene enhances soybean yield potential through modulating plant growth and flowering. *Scientific Reports* **2021,** *11* (1), 12758.

29. Tang, X.; Su, T.; Han, M.; Wei, L.; Wang, W.; Yu, Z.; Xue, Y.; Wei, H.; Du, Y.; Greiner, S.; Rausch, T.; Liu, L., Suppression of extracellular invertase inhibitor gene expression improves seed weight in soybean (Glycine max). *Journal of Experimental Botany* **2017,** *68* (3), 469-482.

30. Chen, L.; Qin, L.; Zhou, L.; Li, X.; Chen, Z.; Sun, L.; Wang, W.; Lin, Z.; Zhao, J.; Yamaji, N.; Ma, J. F.; Gu, M.; Xu, G.; Liao, H., A nodule-localized phosphate transporter GmPT7 plays an important role in enhancing symbiotic N-2 fixation and yield in soybean. *New Phytologist* **2019,** *221* (4), 2013-2025.

31. Preuss, S. B.; Meister, R.; Xu, Q.; Urwin, C. P.; Tripodi, F. A.; Screen, S. E.; Anil, V. S.; Zhu, S.; Morrell, J. A.; Liu, G.; Ratcliffe, O. J.; Reuber, T. L.; Khanna, R.; Goldman, B. S.; Bell, E.; Ziegler, T. E.; McClerren, A. L.; Ruff, T. G.; Petracek, M. E., Expression of the Arabidopsis thaliana BBX32 Gene in Soybean Increases Grain Yield. *Plos One* **2012,** *7* (2), e30717.

32. Liu, S. Y.; Liu, J. F.; Zhang, Y. Z.; Jiang, Y. S.; Hu, S. W.; Shi, A. D.; Cong, Q. Y.; Guan, S. Y.; Qu, J.; Dan, Y., Cloning of the Soybean sHSP26 Gene and Analysis of Its Drought Resistance. *Phyton-International Journal of Experimental Botany* **2022,** *91* (7), 1465-1482.

33. Moretti, L. G.; Crusciol, C. A. C.; Bossolani, J. W.; Momesso, L.; Garcia, A.; Kuramae, E. E.; Hungria, M., Bacterial Consortium and Microbial Metabolites Increase Grain Quality and Soybean Yield. *Journal of Soil Science and Plant Nutrition* **2020,** *20* (4), 1923-1934.

34. Jaybhay, S. A.; Taware, S. P.; Varghese, P., Microbial inoculation of Rhizobium and phosphate-solubilizing bacteria along with inorganic fertilizers for sustainable yield of soybean Glycine max (L.) Merrill. *J. Plant Nutr.* **2017,** *40* (15), 2209-2216.

35. Igiehon, O. N.; Babalola, O. O., Rhizobium and Mycorrhizal Fungal Species Improved Soybean Yield Under Drought Stress Conditions. *Current Microbiology* **2021,** *78* (4), 1615-1627.

36. Mahanta, D.; Rai, R. K.; Mishra, S. D.; Raja, A.; Purakayastha, T. J.; Varghese, E., Influence of phosphorus and biofertilizers on soybean and wheat root growth and properties. *Field Crops Research* **2014,** *166*, 1-9.

37. Mahanta, D.; Rai, R. K.; Dhar, S.; Varghese, E.; Raja, A.; Purakayastha, T. J., Modification of root properties with phosphate solubilizing bacteria and arbuscular mycorrhiza to reduce rock phosphate application in soybean-wheat cropping system. *Ecological Engineering* **2018,** *111*, 31-43.

38. Leite, R. d. C.; Pereira, Y. C.; de Oliveira-Paiva, C. A.; Guedes de Moraes, A. J.; da Silva, G. B., Increase in yield, leaf nutrient, and profitability of soybean co-inoculated with Bacillus strains and Arbuscular mycorrhizal fungi. *Revista Brasileira De Ciencia Do Solo* **2022,** *46*, e0220007.
